# Sequential actions of β-catenin and Bmp pattern the oral nerve net in *Nematostella vectensis*

**DOI:** 10.1038/ncomms6536

**Published:** 2014-12-23

**Authors:** Hiroshi Watanabe, Anne Kuhn, Manami Fushiki, Kiyokazu Agata, Suat Özbek, Toshitaka Fujisawa, Thomas W. Holstein

**Affiliations:** 1 Department of Molecular Evolution and Genomics, Centre for Organismal Studies (COS), Heidelberg University, Im Neuenheimer Feld 329, 69120 Heidelberg, Germany; 2 Department of Biophysics, Graduate School of Science, Kyoto University, Kitashirakawa-Oiwake, Sakyo-ku, Kyoto 606-8502, Japan; 3 Center for the Promotion of Integrated Sciences, The Graduate University for Advanced Studies, Higashiyama 5-1, Myodaiji, Okazaki 444-8585, Japan

## Abstract

Animal evolution is closely linked to the emergence of the nervous system. At present it is unknown how the basic mechanisms of neural induction and formation of central nervous systems evolved. We addressed this question in *Nematostella vectensis*, a member of cnidarians, the ancient sister group of bilaterians. We found that β-catenin signalling is crucial for the early induction of the embryonic nervous system. β-Catenin activity at the blastopore induces specific neurogenic genes required for development of the oral nervous system. β-Catenin signalling induces also Bmp signalling, which, at later larval stages, becomes indispensible for the maintenance and asymmetric patterning of the oral nervous system along the primary and secondary (directive) axes. We hypothesize that the consecutive and functionally linked involvement of β-catenin and Bmp signalling in the formation of the cnidarian oral nervous system reflects an ancestral mechanism that evolved before the cnidarian/bilaterian split.

## Abstract (web summary)

The bilaterian central nervous system is thought to have evolved from a cnidarian-like ancestor, but the mechanisms of neural induction in cnidarians are largely unknown. Here the authors study the cnidarian *Nematostella vectensis* and show that β-catenin signalling is crucial for the early induction of its embryonic nervous system, suggesting evolutionary roots for this pathway.

## Graphical abstract

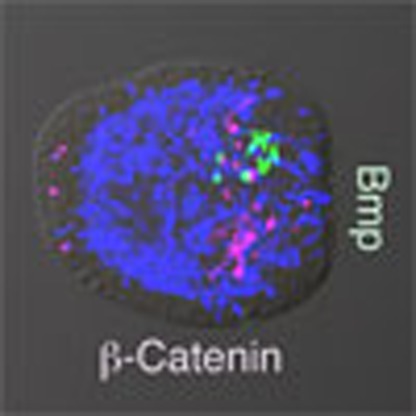

The emergence of nerve cells is one of the key novelties in animal evolution^1^. How cells of an embryo are committed to a neuronal fate is therefore a central question in biology. A related issue is the formation of a central nervous system (CNS), which is coordinating the action of sensory cells and neurons in higher metazoan animals.

The general view holds that the bilaterian CNS can be traced back through evolution to a nerve net in a cnidarian-like ancestor^2^. Cnidarians are simple diploblastic animals with a gastrula-like body plan, and they constitute the sister group of all bilaterians^3^,^4^,^5^,^6^,^7^,^8^,^9^. The cnidarian nervous system is organized as a nerve net with an increased density of neurons at the oral and aboral end of the body axis^5^,^6^,^8^. It also exhibits a remarkable sophistication of sensory cells and organs such as rhopalia^8^. Although the picture of the cnidarian nervous system is not complete, their neurons are known to be rich in neuropeptides, and comparative genomics in distant cnidarian species has revealed an almost complete set of homologous neurogenic transcription factors (TFs) and signalling molecules patterning the nervous system in bilaterians^5^,^8^. Cnidarian neurogenic TFs and neuropeptide-positive neurons exhibit a clear position dependency along the oral–aboral body axis^5^,^6^,^8^,^9^,^10^,^11^,^12^,^13^,^14^. However, the genetic mechanisms controlling the formation of the cnidarian nervous system are largely unknown.

In bilaterians, several secreted factors have been demonstrated to be crucial for the early segregation of the embryonic ectoderm into a neurogenic and a non-neurogenic tissue during neural induction. Bmp signalling is the critical factor for the early development of the CNS and for its anterior–posterior (AP) and dorsal–ventral (DV) patterning. Bmp signalling is suppressed by antagonists such as Chordin and Noggin on the anterior–dorsal side in chordates^15^ and on the ventral side in arthropods, flatworms and other gastroneuralians^16^,^17^,^18^. On the basis of experiments in *Xenopus* and *Drosophila*, which showed that an inhibition of Bmp signalling is sufficient to induce the neuroectoderm, it was proposed that the primary function of Bmp signalling in bilaterians is the inhibition of the neural fate, and accordingly, the default state of the ectoderm would be the neural fate^15^,^16^,^19^,^20^. This hypothesis was questioned for vertebrates because mutants lacking Bmp antagonists still develop neurons^21^. Also in other deuterostomes and in annelids, perturbation of Bmp signalling did not change the expression of neural makers^22^,^23^,^24^. Therefore, Bmp/Chordin signalling is not necessarily linked to neural induction in bilaterians^21^.

Wnt/β-catenin signalling has multiple and even opposite functions in neural development of deuterostomes, depending on the developmental stage^21^. Canonical Wnt signalling is required for neurogenesis at the dorsal side of early chordate embryos and for the AP and DV patterning of the CNS^21^. At later stages of chordate development, the pathway has a function in the posteriorization of the neural ectoderm^26^, which is similar to hemichordates and echinoderms^27^,^28^. The neurogenic function of Wnt signalling for the transition of proliferating neuronal precursor cells to differentiating neurons in deuterostomes^25^ has been recently described in lophotrochozoans^29^. Thus, a neurogenic function of Wnt/β-catenin signalling might be an ancestral bilaterian trait^29^. To understand which signalling pathway(s) controls neurogenesis in cnidarians, we focused our analysis on the signalling pathways and TFs that control the formation of the oral nervous system in *N. vectensis*. Many cnidarian polyps develop a conspicuous ring-like nerve plexus around their mouth^8^,^10^,^30^. This cnidarian nerve ring was repeatedly depicted as a beginning of the bilaterian CNS^10^,^31^,^32^.

The first sign for neurogenesis in *N. vectensis* is the salt-and-pepper-like expression of neural marker messenger RNAs (mRNAs) such as *NvAchaete-scute homolog A* (*NvAshA*), *NvElav1* and *Rfamide* in blastula epithelium^6^,^13^,^14^. Recent studies provided evidence for their involvement in neural development of *N. vectensis* embryos. A whole-genome microarray analysis on *NvAshA*-overexpressing embryos has identified *NvElav1*, *NvRfamide* and genes involved neurotransmission as *NvAshA* targets^13^. It was demonstrated that *NvElav1* is required at least ectodermal development of neuropeptides-expressing neurons^16^. The pervasive expression of *NvAshA* suggests that the early embryonic epithelium of *N. vectensis* has the potential to generate various neuronal cell types that form the larval nerve net^13^. In addition to this pervasive expression of neural genes, the presumptive blastopore region of the blastula expresses *NvAshB*, which is also involved in *NvRfamide* neuropeptide gene expression^13^. This suggests that the blastoporal side of the early embryos has distinct neurogenic feature(s). The blastopore of the gastrula in fact develops into a prominent neurogenic domain at the planula stage, which is characterized by some neural markers, *NvAshC*, *NvSoxC*, *NvMusashi* and *NvRfamide* that are expressed dominantly or exclusively around the blastopore^6^,^12^,^13^,^14^,^33^. This oral neurogenic domain of the embryo develops into an elaborate nerve plexus at the oral side of the planula larva and primary polyp that comprises a number of subsystems with separate physiological properties^8^.

Our study revealed that β-catenin signalling is essential for early neurogenesis during the development of the oral nervous system that starts at the blastula/gastrula transition. Wnt/β-catenin signalling is known to be active at the blastoporal side and defines the primary oral–aboral axis in *N. vectensis* blastulae^34^,^35^. By comparison, Bmp2/4 is also expressed at the blastoporal side, however, with a strong bias towards a secondary ‘directive’ body axis at later development^36^,^37^,^38^. We also show that in a subsequent developmental phase Bmp signalling has crucial influences on the regionalized development of the nervous system along both the primary and secondary (directive) body axes. Our data indicate that the sequential action of β-catenin and Bmp signalling in the cnidarian *N. vectensis* reflects the evolutionary emergence of these major signalling axes in the evolution of the nervous system.

## Results

### Early development of neuropeptide-positive neurons

The cnidarian nervous system is rich in neuropeptides^8^,^11^,^39^. Among these, the short amidated neuropeptides RFamide and GLWamide, belonging to R[F/Y]amide and [G/V/L]Wamide groups, respectively, are known to have deep evolutionary roots in the common *Cnidaria/Bilateria* ancestor^40^. These neuropeptides serve as specific markers for mature neurons in cnidarians^8^, and in bilaterians they are expressed in neuronal subpopulations of the CNS^41^,^42^.

**Figure 1:**
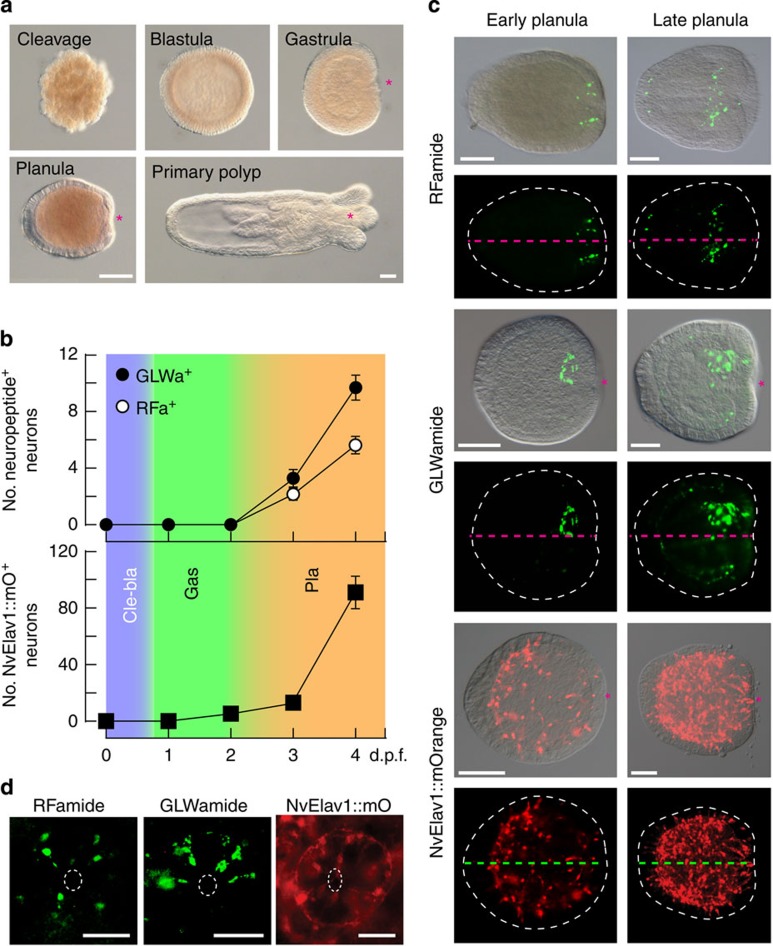
An asymmetry in the *N. vectensis* nervous system. (**a**) Development of *N. vectensis* from cleavage to the primary polyp (differential interference contrast images). (**b**) Number of neurons expressing the mature neuropeptides RFamide and GLWamide (upper graph), and number of NvElav1::mOrange positive neurons (lower graph) at different days post fertilization (d.p.f.). Colours indicate cleavage/blastula (Cle-bla), gastrula (Gas) and planula (Pla) stage. The data represent the mean±s.e.m. of at least three experiments (*n*=486 RFamide, *n*=423 GLWamide and *n*=239 Elav). (**c**,**d**) Lateral view (**c**) and oral view (**d**) of RFa^+^, GLWa^+^ and NvElav1::mOrange^+^ neurons in planula larvae. The pink and green dotted lines in **c** denote the oral–aboral axis and the dotted white circles in **d** indicate the blastopore. Note that only GLWa^+^ neurons exhibit an additional bias along the orthogonal axis around the blastopore. Shown are representative images of at least three with similar results. The asterisks in **a** and **c** indicate the blastopore. Scale bars in **a**,**c** and **d**, 100 μm and 50 μm, respectively.

We studied various developmental stages of *N. vectensis* that belongs to anthozoans, the most basal class in cnidarians (Fig. 1a)^7^,^43^,^44^, and is known to show ‘bilateral organization of the endoderm^45^. Analyses using antibodies specific for the mature form of RFamide and GLWamide neuropeptides demonstrated that functional and peptidergic neurons are already present in early planulae (Fig. 1b,c)^6^,^14^. It has also recently been shown that *NvElav1* is expressed in a substantial part of neurons during embryogenesis^6^,^14^. An NvElav1^+^ neuron-specific transgenic reporter line, where the mOrange fluorescent protein is expressed under the *NvElav1* regulatory elements, demonstrated the development of the NvElav1::mOrange^+^ neurons at gastrula and planula stages^14^. A quantitative analysis revealed that the RFamidergic (RFa^+^) and GLWamidergic (GLWa^+^) neuronal subpopulations correspond to 10% of all neurons at the late planula stage (Fig. 1b). The RFa^+^ neurons develop in the entire ectoderm and form an elaborated nerve plexus at the oral side, but they did not form an aboral sensory cluster (Fig. 1c)^6^,^14^, which is often observed in planula larvae of hydrozoans^46^. In late planulae and after metamorphosis into a primary polyp, a domain rich in RFa^+^ perikaria was formed in the blastoporal and the hypostomal/tentacle region (Fig. 1c; Supplementary Fig. 1)^6^. GLWa^+^ neurons differentiate in the lateral ectoderm and in the oral endoderm of early planulae (Fig. 1d; Supplementary Fig. 2). The endodermal GLWa^+^ neurons formed a neuronal cluster in an asymmetric manner on one side at the oral region of the planula larvae (Fig. 1c,d), whereas the ectodermal neurons are distributed symmetrically and mainly in the midst body region (Supplementary Fig. 2). The asymmetry of the endodermal cluster of GLWa^+^ neurons completely vanished in primary polyps (Supplementary Fig. 1). The spatial arrangement of neurons at the oral region is not unique to the peptidergic neurons because Elav^+^ neurons form a ring-like sensory cell cluster around the blastopore at late planula stage (Fig. 1d). The formation of RFa^+^ and GLWa^+^ neurons at the oral side of early planula larvae suggests that those precursors were generated directly at the blastoporal region in early developmental stages, for example, gastrula. We therefore analysed the TFs expressed at the blastopore region and signalling pathways establishing the oral nervous system.

### Identification and expression of oral neurogenic TFs

**Figure 2:**
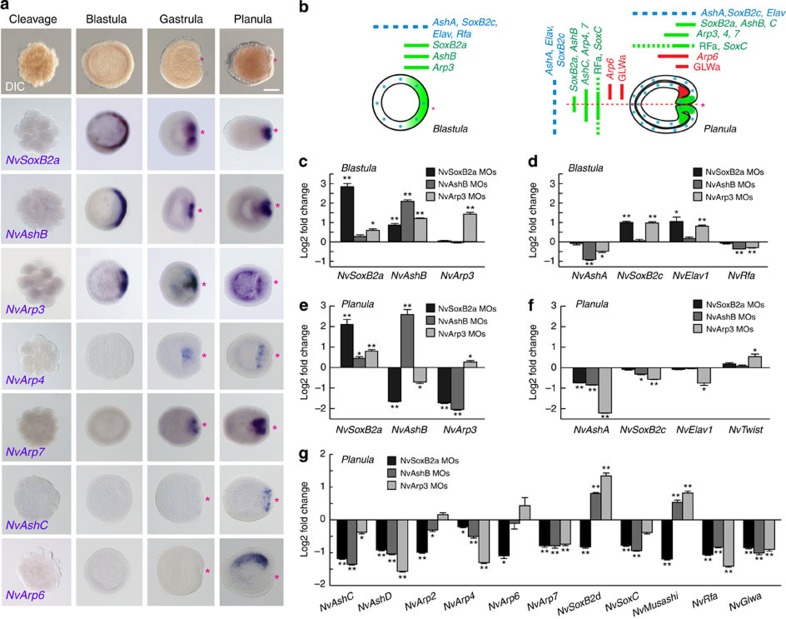
Action of early oral neurogenic genes. (**a**) WISH assay of neural transcription factors. Red asterisks denote the blastopore and future oral pole. Shown are representative images of at least three with similar results. Note the late asymmetric expression of *NvArp6* at the planula stage. Scale bar, 100 μm. (**b**) A schematic illustration of the expression patterns of oral and lateral neurogenic and marker genes at the blastula (left) and planula (right) stages. (**c**–**g**) qPCR analyses of the neural gene expression in the blastula (**c**,**d**) and planula (**e**,**f**) that were injected with MOs against *NvSoxB2a*, *NvAshB* or *NvArp3*. Bars represent the mean±s.e.m. of at least three experiments. The black asterisks denote the statistical significance using the Student’s *t*-test (*P<0.05, **P<0.01).

We screened the EST and genomic database of *N. vectensis* for TFs that have conserved neurogenic functions among bilaterians. We determined the temporal and spatial expression patterns of the TFs during *N. vectensis* embryogenesis and analysed their neurogenic activity. Our reverse transcriptase (RT)-PCR and whole-mount *in situ* hybridization (WISH) data demonstrate that several neurogenic genes were strongly upregulated during early embryonic development (Fig. 2a,b; Supplementary Fig. 3). We found that *NvAtonal*-related protein 3 (*NvArp3*) formed a synexpression group at the prospective and established blastopore site together with the previously described *NvSoxB2a* and *NvAshB*^12^,^13^ during the blastula and gastrula stage (Fig. 2a,b). *NvSoxB2a* was considered to be an unclassified *NvSox1* (ref. 12), but has recently been identified as the orthologous gene to the bilaterian *SoxB2* genes and was reclassified as *NvSoxB2* (ref. 47). Since the C-terminal region of NvSoxB2a is similar to bilaterian SoxB2 (Supplementary Fig. 4), NvSoxB2a might have a conserved neurogenic function^47^. At the planula larva stage, *NvSoxB2a* and *NvAshB* were restricted to the oral side of the pharynx, while *NvArp3* was expressed in cells distributed broadly at the endodermal layer. The concurrent expression of *NvSoxB2a* with *bHLH* genes in *N. vectensis* is in accord with results from bilaterians, where *SoxB2* genes are expressed in neural precursors together with *bHLH* genes^48^. We identified additional bHLH TFs showing also distinct blastoporal expression patterns at the oral region, that is, *NvArp4* in endoderm, *NvArp7* in pharynx and *NvAshC* in ectoderm^13^. Furthermore, we were able to find *NvArp6* as the first neurogenic TF being asymmetrically expressed along the secondary (directive) axis (Fig. 2a,b).

We next analysed the genetic relationships between these early oral neurogenic genes (that is, *NvSoxB2a*, *NvAshB* and *NvArp3*) and other early neurogenic and neural marker genes (*NvAshA*, *NvSoxB2c* (former SoxB2), *NvElav1* and *NvRfamide*) as well as the late oral neurogenic genes (*NvAshC*, *D*, *NvArp2*, *4*, *6*, *7*, *NvSoxB2d*, *C*, *NvMusashi*, *NvRfamide* and *NvGlwamide*) using morpholino (MO) antisense oligonucleotides against the early blastoporal neurogenic genes (Supplementary Fig. 5). Figure 2c,d show that in morphants of *NvSoxB2a*, *NvAshB* and *NvArp3* an upregulation of all early genes at the blastula stage, except for *NvAshA* and *NvRfamide*, indicating that *NvSoxB2a*, *NvAshB* and *NvArp3* are not required for the initial steps of most of these genes, but rather form a negative gene regulatory circuit. At the planula stage, however, a suppression of *NvSoxB2a* resulted in the downregulation of *NvAshB* and *NvArp3* (Fig. 2e), indicating that *NvSoxB2a* maintains the expression of these early oral bHLH genes. At the planula stage, expression of the lateral genes (Fig. 2f) and the late oral genes (that is, neurogenic TFs and neuropeptides) (Fig. 2g) were affected in these morphants. This clearly demonstrated the crucial function of early oral neurogenic genes for the development of the oral nervous system. The MO experiments indicated also that these early oral neurogenic TFs are involved at least partially in the transcriptional control of lateral neural markers from the blastula to planula stage (Fig. 2d,f). The function of the early oral genes is specific to neurogenesis because expression of *NvTwist*, a marker for endoderm was not suppressed in these morphants (Fig. 2f).

**Figure 3:**
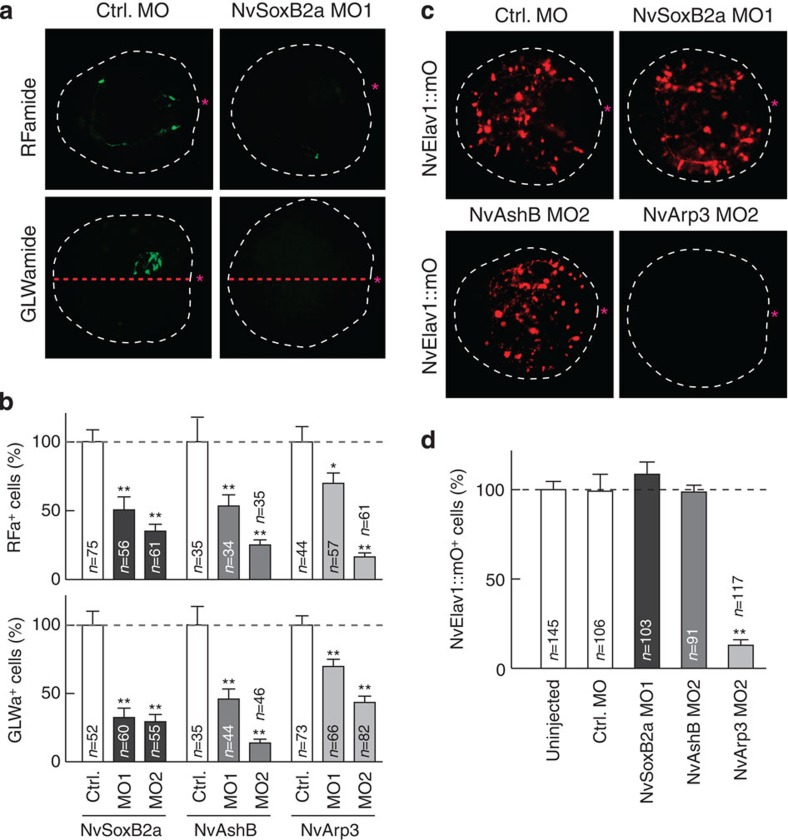
Oral neurogenic genes are required for RFa^+^, GLWa^+^ and Elav1^+^neurons. Development of RFa^+^ and GLWa^+^ neurons (**a**,**b**) and NvElav1::mOrange^+^ neurons (**c**,**d**) in the planula injected with MO against *NvSoxB2a*, *NvAshB* and *NvArp3*. (**a**) RFa^+^ and GLWa^+^ neurons in control MO or NvSoxB2a MO-injected planulae (3 d.p.f.). The red dotted lines denote the oral–aboral axis. (**c**) NvElav1::mOrange^+^ neurons in MO-injected planulae (3 d.p.f.). The red asterisks indicate the blastopore. (**b**,**d**) Number of RFa^+^ and GLWa^+^ neurons (4 d.p.f.) (**b**) and NvElav1::mOrange^+^ neurons (3 d.p.f.) (**d**). The data in **b** and **d** represent the relative number of neurons. Bars represent the mean±s.e.m. The black asterisk denotes the statistical significance using the Student’s *t*-test (*P<0.05, **P<0.01).

We further analysed the neurogenic activity of the early oral TFs by monitoring the mature RFa^+^ and GLWa^+^ neurons. For all morphants, we confirmed that these genes are required for ectodermal and endodermal development of RFa^+^ and GLWa^+^ neurons (Fig. 3a,b; Supplementary Figs 6 and 7). Interestingly, development of NvElav1::mOrnage^+^ neurons were inhibited only in *NvArp3* MO-injected planula larvae (Fig. 3c,d), which is consistent with the specific involvement of *NvArp3* on *NvElav1* expression at the planula stage (Fig. 2f). These results clearly indicate that *NvSoxB2a*, *NvAshB* and *NvArp3* have neurogenic function and that the presumptive blastopore region of the blastula represents an early neurogenic field, which gives rise to neuronal populations of the oral (RFa^+^/GLWa^+^) and endodermal (*NvElav1*^+^) nervous systems. As *NvSoxB2a* and *NvAshB* are required for the expression of many early and late oral neuronal genes, they might mainly be involved in the commitment of embryonic cells that gives rise to neural precursor cells, whereas *NvArp3* might have a selective function to specify certain neuronal cell types such as Elav1^+^ neurons.

### β-Catenin signalling in early neurogenesis

To identify signals required for expression of the early oral neurogenic genes, we searched for factors turned on in the blastula at the site of presumptive blastopore region. RT-PCR and WISH analyses indicated that *NvBrachyury* (*NvBra*), a target of canonical Wnt/β-catenin signalling in cnidarians^49^, is strongly upregulated at the blastula stage (Supplementary Fig. 8). Although β-catenin protein seems to be expressed in all blastomeres at the early-cleavage stages, it has been described to become stabilized and translocated to the nuclei at the embryonic region where gastrulation will occur^34^ and *NvTcf* expression is maintained (Supplementary Fig. 8)^50^. Wnt/β-catenin activity is maintained in later developmental stages (planula and polyp) at the blastopore/mouth^35^,^50^ and was postulated to pattern gene expression (including *NvRfamide*) along the oral–aboral (primary) body axis^51^,^52^. The β-catenin function on the early phase of neurogenesis, however, has not yet been clarified. The concurrent temporal and spatial expression pattern of early neurogenic genes with downstream target genes of the β-catenin pathway at the blastula stage prompted us to investigate the involvement of β-catenin signalling in the early development of the oral nervous system.

**Figure 4:**
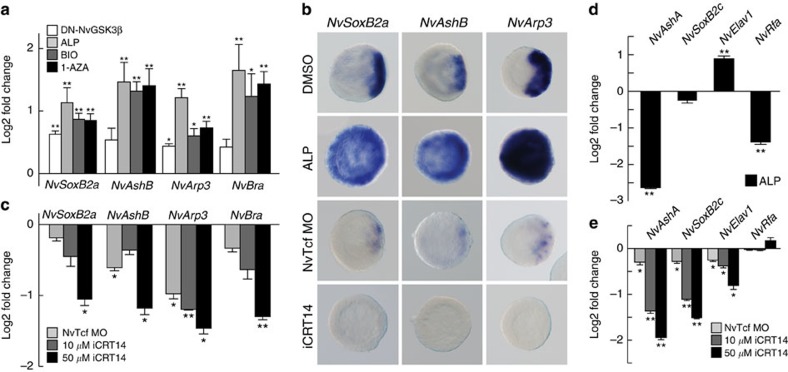
β-Catenin signalling regulates early neurogenesis. (**a**-**c**) Expression analyses of early neurogenic genes *NvSoxB2a*, *NvAshB* and *NvArp3* at the blastula stage after DN-GSK3β mRNA injection or treatment with several GSK3β inhibitors (2.5 μM ALP, 0.5 μM BIO and 2.5 μM 1-AZA) (**a**,**b**) and in blastulae injected with NvTcf MO or treated with an increasing concentrations of iCRT14 (**b**,**c**). *NvBra* was used as positive control in the qPCR analyses (**a**,**c**). In the WISH analysis (**b**), ALP and iCRT14 were used at 2.5 μM and 50 μM concentrations, respectively. (**d**,**e**) qPCR analyses of early lateral neural genes *NvAshA*, *NvSoxB2c*, *NvElav1* and *NvRfamide* in 2.5 μM ALP-treated blastulae (**d**), in Tcf morphants and in 50 μM iCRT14-treated blastulae (**e**). Shown in **b** are representative images of at least three with similar results. Bars represent the mean±s.e.m. of at least three experiments. Asterisks denote statistical significance using the Student’s *t*-test (*P<0.05; **P<0.01).

To examine the function of the β-catenin pathway in the neural induction, we stimulated β-catenin signalling by injecting mRNA for a dominant-negative GSK3-β (DN-GSK3β) into fertilized eggs. The augmentation of β-catenin signalling induced increased expression of the neurogenic genes *NvSoxB2a*, *NvAshB* and *NvArp3*, as well as a β-catenin target gene *NvBra* at the blastula stage (Fig. 4a). A more prominent and ectopic upregulation of neurogenic gene expression could be observed when early embryos were treated with the GSK3-β inhibitors Alsterpaullone (ALP), BIO or 1-Azakenpaullone (1-AZA) (Fig. 4a,b; Supplementary Figs 8b and 9). To analyse β-catenin regulation of the neurogenic gene expression at early embryonic stages, we treated embryos only for a short time (that is, from the egg to the blastula stage). The temporal activation of β-catenin signalling with GSK3β inhibitors was also needed to circumvent potential toxic effects caused by a long-term treatment with these inhibitors^51^, so that the mortality rate of planulae remained unchanged (Supplementary Fig. 10). Under these conditions, we observed an ‘oralized’ phenotype with an ectopic expression of early neurogenic TFs (Fig. 4; Supplementary Fig. 8b). In blastula embryos, the injection of NvTcf MO or treatment of Tcf/β-catenin inhibitor iCRT14 (ref. 53) suppressed the expression of *NvSoxB2a*, *NvAshB* and *NvArp3* (Fig. 4b,c). These data therefore indicate that the β-catenin pathway positively regulates expression of the oral neurogenic TFs at the blastula stage.

Next, we analysed the β-catenin regulation of early genes, *NvAshA*, *NvSoxB2c*, *NvElav1* and *NvRfa*mide that show lateral expression patterns at blastula. Most of these genes except for *NvElav1* were not upregulated under augmented β-catenin signalling (Fig. 4d), whereas *NvAshA*, *NvSoxB2c* and *NvElav1* were strongly suppressed by the β-catenin inhibitor (Fig. 4e). These data indicate that β-catenin signalling is globally required at an early developmental stage for the expression of oral and lateral neural genes and neurogenic TFs.

**Figure 5:**
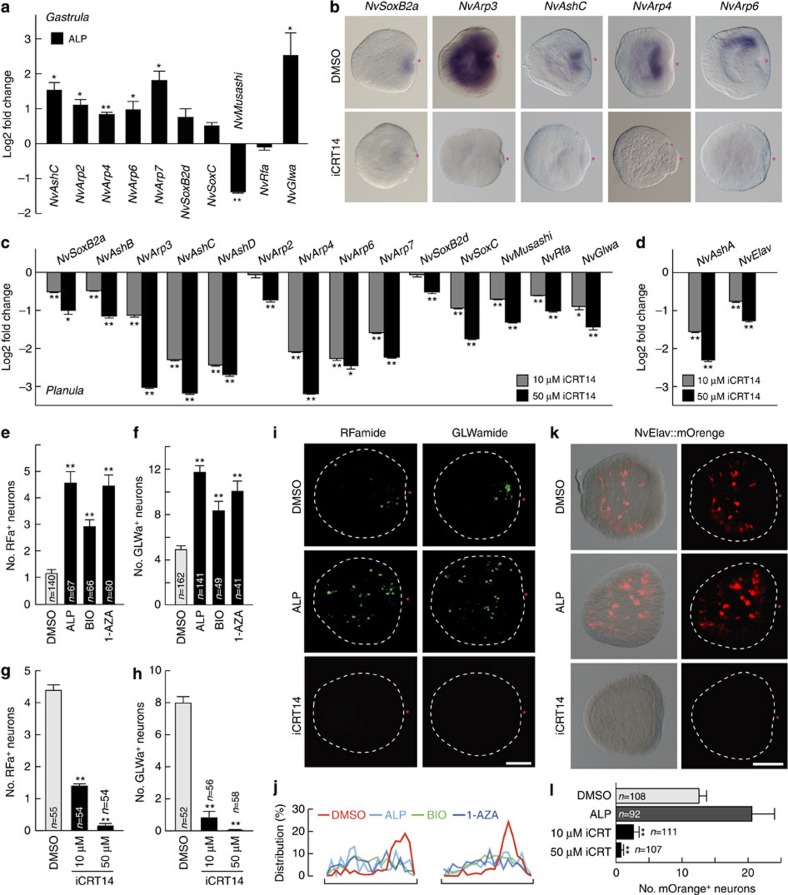
β-Catenin signalling positions neuronal genes to the oral site. (**a**) qPCR analysis of late oral neural genes in 2.5 μM ALP-treated gastrula (16 h.p.f.). (**b**) WISH analysis of oral neurogenic transcription factors in the planula treated without (dimethyl sulfoxide (DMSO)) or with 50 μM iCRT14. (**c**,**d**) qPCR analyses of the oral and lateral neural gene expression in the iCRT14-treated planulae. (**e**–**h**) Quantification of number of RFa^+^ (**e**,**g**) and GLWa^+^ (**f**,**h**) neurons in the planula larvae treated with GSK3β inhibitors (2.5 μM ALP, 0.5 μM BIO or 2.5 μM 1-AZA) up to the blastula stage (**e**,**f**) or treated with increasing concentrations of iCRT14 up to the planula stage (**g**,**h**). Neurons were counted at 3 d.p.f. (**e**,**f**) or 4 d.p.f. (**g**,**h**). (**i**–**l**) Development of the RFa^+^ and GLWa^+^ neurons (**I**,**j**) and NvElav1::mOrange^+^ neurons (**k**,**l**) in the planula treated with 2.5 μM ALP (up to blastula) or 50 μM iCRT14. (**j**) Distributions of RFa^+^ (left) and GLWa^+^ (right) neurons along the OA axis of planulae treated with GSK3β inhibitors (2.5 μM ALP, 0.5 μM BIO or 2.5 μM 1-AZA) up to the blastula stage were plotted (*n*=9 (ctrl.), *n*=9 (ALP), *n*=6 (BIO) and *n*=6 (1-AZA) for RFa; *n*=21 (ctrl.), *n*=10 (ALP), *n*=9 (BIO) and *n*=7 (1-AZA) for GLWa). (**l**) Quantification of number of NvElav1::mOrange^+^ neurons in the planula larvae treated with 2.5 μM ALP increasing concentrations of iCRT14. Shown in **i** and **k** are representative images of at least three with similar results. Scale bars, 100 μm. The red asterisks in **b**,**i** and **k** denote the blastopore. Bars represent the mean±s.e.m. of three experiments. Black asterisks denote statistical significance using the Student’s *t*-test (*P<0.05; **P<0.01).

The wide spectrum of β-catenin dependency of neurogenic TFs suggests that rather for specifying certain neuronal cell types, β-catenin signalling is required for the early fate decision of orally and laterally localized neural progenitors giving rise to multiple neuronal cell types in the planula nervous system. Consistent with this idea, the activation of β-catenin signalling at early embryogenesis (egg-blastula) also induced strong upregulation of other neural genes that show a later oral expression at the gastrula stage (Fig. 5a). All neurogenic and neural marker genes analysed were suppressed at planula stage after the iCRT14 inhibitor treatment (Fig. 5b–d). The early stage-specific activation of the β-catenin pathway resulted in an ectopic development and increase in the number of both, RFa^+^ and GLWa^+^ neurons at the planula stage (Fig. 5e,f,i,j; Supplementary Fig. 11). The iCRT14 treatment confirmed the β-catenin requirement for the development of RFa^+^, GLWa^+^ and Elav::mOrange^+^ neurons (Fig. 5g–i,k,l). Thus, global and localized actions of β-catenin signalling are essential for the neural commitment of early embryonic cells and for the formation of a region with a high neurogenic potential that then develops into the oral nervous system.

### Bmp signalling in late neural patterning at the oral side

**Figure 6:**
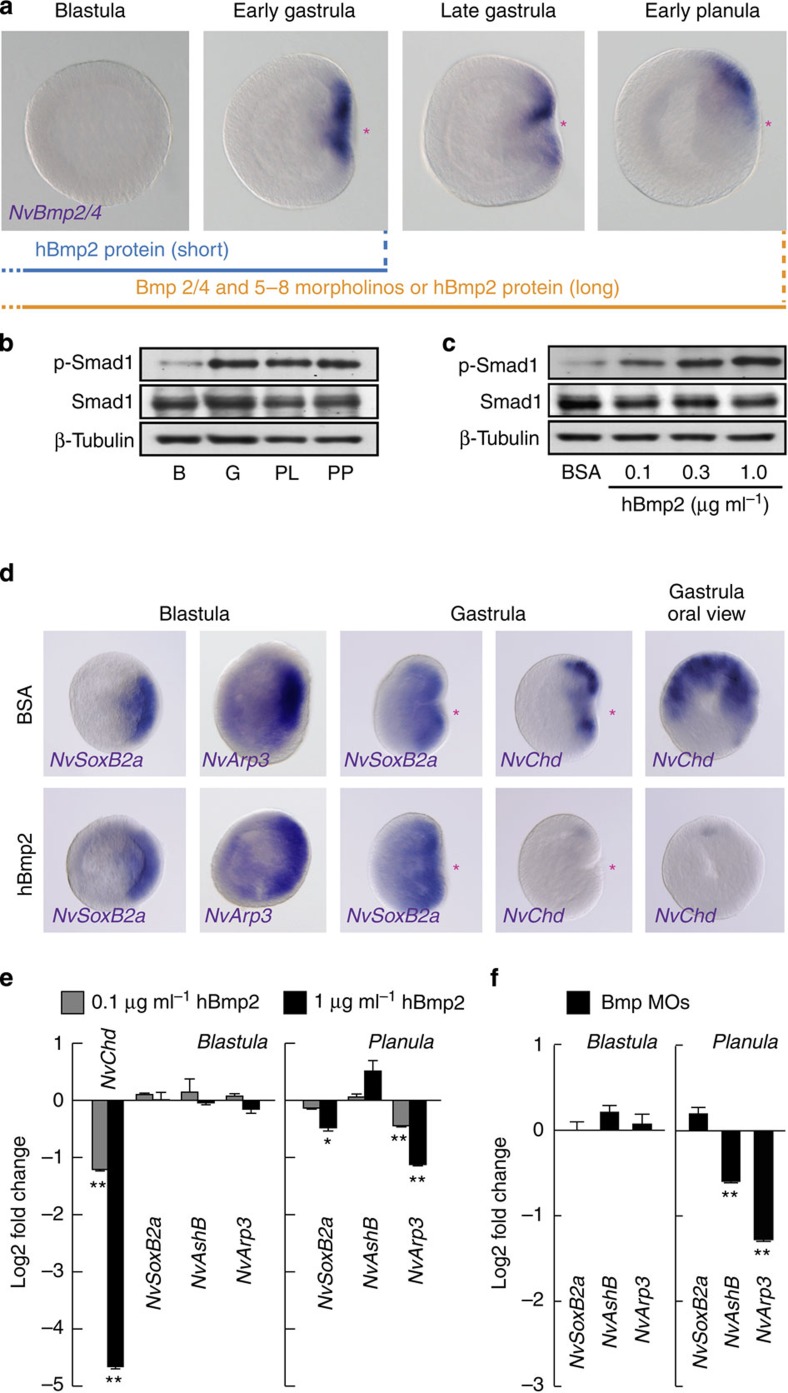
Bmp-independent early neurogenesis. (**a**) WISH analysis of orally expressed *NvBmp2/4* and scheme of hBmp2 treatment and of knockdown using morpholinos against Bmp2/4 and Bmp5–8. (**b**,**c**) NvSmad1 phosphorylation at the blastula (B), gastrula (G), planula (P) and primary polyp (PP) stages (**b**) and in blastula treated with increasing concentrations of exogenous hBmp2 protein (**c**). Smad1 and β-tubulin proteins were used as internal control. (**d**) WISH analysis of the effect of hBmp2 treatment (1 μg ml^−1^) on the expression of *NvSoxB2a*, *NvArp3 and NvChordin* (*NvChd*). (**e**) qPCR analysis of the early neurogenic genes at the blastula (left) and planula (right) treated with increasing concentrations of hBmp2. (**f**) qPCR analysis of early neurogenic genes at the blastulae and planula larvae injected with MOs against *NvBmp2/4* and *NvBmp5–8*. Red asterisks denote the blastopore and future oral pole. Bars represent the mean±s.e.m. of three experiments. Black asterisks denote statistical significance using the Student’s *t*-test (*P<0.05; **P<0.01).

We next analysed the effect of Bmp signalling on the development of the oral nervous system in *N. vectensis*. The asymmetric expression of *NvBmp2/4*, *NvBmp5–8* and their antagonist *NvChordin* became visible around the blastoporal lip only after the gastrula stage (Fig. 6a)^37^,^38^,^54^. Although an early expression of *NvBmp2/4* and *NvChordin* was reported at the blastula stage by RT-PCR and microarray analyses^37^,^55^, no regionalized expression was detectable at this stage. The oral expression of *NvBmp2/4*, *NvBmp5–8* and *NvChordin* was depending on the β-catenin signalling, as the expression was suppressed by iCRT14 (Supplementary Fig. 12). An augmentation of β-catenin signalling by 1-AZA induced upregulation of *NvBmp2/4* and *NvBmp5–8* expression, whereas *NvChordin* expression was inhibited, suggesting that the dual role of β-catenin signalling on the *NvChordin* expression. When we analysed the level of activated Bmp signalling by determining phosphorylation levels of the NvSmad1 C terminus, we found a strong signal at the gastrula stage (Fig. 6b), suggesting that the local expression of *NvBmp2/4* and *NvBmp5–8* in the gastrula is required for an activation of Bmp signalling. To test whether Bmp signalling functions in the delimited expression of early neurogenic TFs at the prospective blastoporal side of the blastula, we exposed early embryos to increasing concentrations of exogenous human Bmp2 protein (hBmp2) to globally elevate the Bmp pathway. hBmp2 treatment induced the phosphorylation of NvSmad1 in a dose-dependent manner (Fig. 6c). The expression of *NvChordin* was strongly suppressed by the augmented Bmp signalling (Fig. 6d)—as has been demonstrated in bilaterians and *N. vectensis*^36^—whereas the polarized expression of early oral neurogenic TFs at the future oral side of the blastula and gastrula was not altered (Fig. 6d). Consistently, development of the RFa^+^ and GLWa^+^ neurons was not affected by the early hBmp2 treatment (Supplementary Fig. 13). The hBmp2 suppression of *NvChordin* in the early embryos was confirmed by quantitative PCR (qPCR), whereas no significant effect was observed on the expression level of the early oral TFs (Fig. 6e). Elimination of Bmp signalling by co-injection of MOs for *NvBmp2/4* and *NvBmp5–8* (ref. 36) confirmed that the expression of the early neurogenic TFs is independent of Bmp signalling at the blastula stage (Fig. 6f).

**Figure 7:**
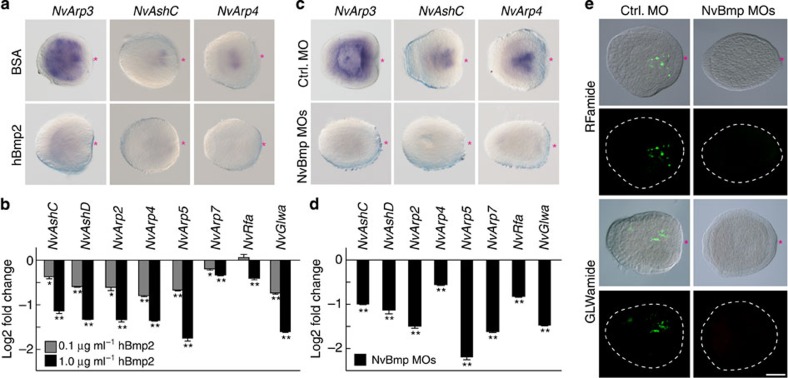
Bmp-dependent late neurogenesis. (**a**–**d**) Expression analyses of oral neurogenic genes in the planulae treated with hBmp2 protein (**a**,**b**) or injected with MOs against *NvBmp2/4* and *NvBmp5–8* (**c**,**d**). WISH analysis shows the expression *NvArp3*, *NvAshC* and *NvArp4* after hBmp2 (1.0 μg ml^−1^) treatment (**a**) or after Bmp MOs injection (**c**) at the planula stage. qPCR analyses show the expression of the oral neural genes at the planula larvae treated with increasing concentrations of hBmp2 (**b**) or injected with Bmp MOs (**d**). (**e**) Development of the RFa^+^ and GLWa^+^ neurons in NvBmp morphants. Shown in **a**,**c** and **e** are representative images of three experiments with similar results. Red asterisks indicate the blastopore. Scale bars, 100 μm. Bars represent the mean±s.e.m. of at least three experiments. Black asterisks denote statistical significance using the Student’s *t*-test (*P<0.05; **P<0.01).

Finally, we extended the hBmp2 treatment up to the planula stage to test the effect of Bmp signalling on TF genes that are also expressed in late neurogenesis. Surprisingly, under these conditions, the expression of early oral *NvArp3* and most of the late oral neurogenic TFs as well as *NvGlwamide* were largely reduced by exogenous hBmp2 in a concentration-dependent manner (Figs 6e and 7a,b). When we further analysed the expression of the same set of genes in Bmp morphants, we also observed a significant suppression of these early and late oral neurogenic genes and of *NvRfamide* and *NvGlwamide* (Figs 6f and 7c,d), which resulted in a decreased number of RFa^+^ and GLWa^+^ neurons at planula stage (Fig. 7e; Supplementary Fig. 14). These data indicate that Bmp signalling is positively and negatively involved in the development of the oral nervous system. This dual function of Bmp signalling is reminiscent to the Bmp-dependent transcriptional regulation of other oral genes^36^.

### NvArp6 induces the asymmetric pattern of NvGLWa^+^ neurons

**Figure 8:**
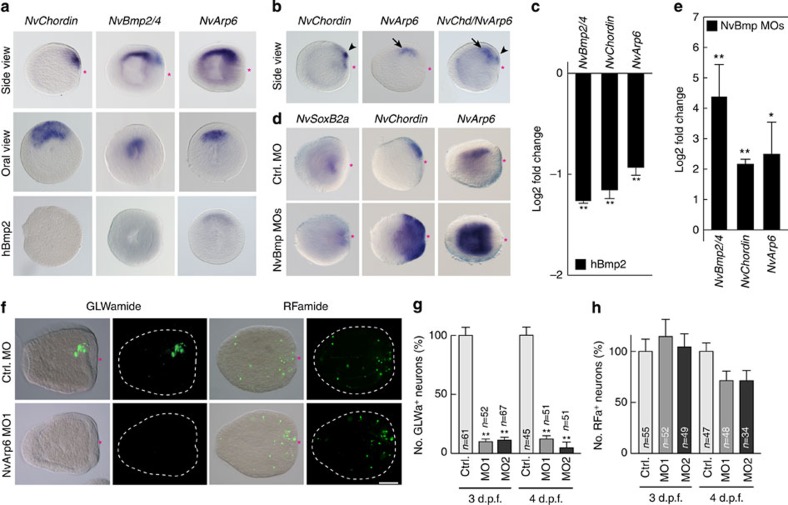
Asymmetric differentiation of GLWa^+^ neurons by Bmp and NvArp6. (**a**–**e**) WISH (**a**,**b**) shows the expression of *NvChordin*, *NvBmp2/4* and *NvArp6* on the same site along the directive axis at the planula stage. Arrowheads and arrows in **b** indicate expression of *NvChordin* and *NvArp6*, respectively. Influence of 1 μg ml^−1^ hBmp2 treatment on the expression of *NvArp6*, *NvBmp2/4* and *NvChordin* is shown in the lower panel of **a**. (**c**) qPCR analysis of *NvBmp2/4*, *NvChordin* and *NvArp6* in planulae treated with 1 μg ml^−1^ hBmp2. (**d**) WISH analysis of *NvSoxB2a*, *NvChordin* and *NvArp6* expression in Bmp morphants. (**e**) qPCR analysis of *NvBmp2/4*, *NvChordin* and *NvArp6* in Bmp morphants. Bars in **c** and **e** represent the mean±s.e.m. of three experiments. (**f**–**h**) *NvArp6*-dependent asymmetric formation of GLWa^+^ neurons. Immunocytochemical analysis of GLWa^+^ and RFa^+^ neurons (**f**) and quantification of GLWa^+^ (**g**) and RFa^+^ (**h**) neuron formation in *NvArp6* morphants. The data in **g** and **h** represent the relative number of neurons. The bars represent the mean±s.e.m. Black asterisks denote statistical significance (*P<0.05; **P<0.01). Shown in **a**,**b**,**d** and **f** are representative images of three experiments with similar results. Red asterisks indicate blastopore. Scale bars, 100 μm.

Among the TFs with a blastoporal expression pattern, *NvArp6* was exceptional in that it exhibits a distinct asymmetric pattern along both body axes, that is, the oral–aboral and the directive axis (Fig. 8a). Double WISH analyses indicated that *NvArp6* is expressed at the same side of the directive axis as *NvChordin* (Fig. 8b), and *NvBmp2/4* and *NvBmp5–8* (ref. 37). Different from *NvChordin*, which was restricted to the oral ectoderm, the gradient of *NvBmp2/4*, *NvBmp5–8* and *NvArp6* expression along the primary axis was reaching the aboral side of endoderm. It was proposed that the overlapping expression of the *NvBmp* ligands and its antagonist *NvChordin* on the same side of the embryo creates a complex gradient of Bmp activity along the primary and directive axes^36^. Accordingly, the overlapping expression domains of *NvBmp2/4* and *NvArp6* indicate a Bmp-mediated suppression of *NvArp6* transcription. To test this hypothesis, we either activated or suppressed Bmp signalling and determined *NvArp6* expression levels at the planula stage. *NvArp6* expression was significantly suppressed by hBmp2 treatment and activated in NvBmp2/4/5–8 double morphants (Fig. 8a,c–e). Note that the *NvSoxB2a* was left unchanged in the Bmp-depleted planula larvae (Fig. 8d). These data clearly indicate that Bmp signalling has a suppressive function in inducing *NvArp6* expression along the primary and directive axes.

We next analysed whether the asymmetric development of the GLWa^+^ neuronal subset along the directive axis was NvArp6 dependent. In NvArp6 morphants, we found an almost complete inhibition of GLWa^+^ neurons (Fig. 8f), whereas RFa^+^ neurons were not affected. A quantitative analysis using NvArp6 morphants confirmed this principal finding (Fig. 8g,h). These data clearly show that NvArp6 activity is specifically required for differentiation of GLWa^+^ neurons. Our data also indicate that the localized induction of *NvArp6* by Bmp suppression demarcates a specific neurogenic region in the oral nervous system along directive axis to give rise to the GLWa^+^ neuronal subdomain.

## Discussion

Here, we analysed neural development in embryos of the cnidarian sea anemone *N. vectensis*. We focused our study on the genetic mechanisms neuralizing embryonic cells and regulating the development of the oral nervous system, which forms a conspicuous nerve plexus around the mouth of many polyps^8^,^10^,^30^ and was repeatedly depicted as the precursor of the bilaterian CNS^10^,^31^,^32^. We used neuronal markers that are abundantly or exclusively expressed during the early development of the oral nervous system at the side of the blastopore of the embryo and planula larva^8^,^12^,^13^,^14^,^33^. By deciphering the consecutive actions of β-catenin and Bmp signalling in the development of the oral nervous system in *N. vectensis* embryos, our findings replicate the evolutionary emergence of these major signalling axes in animal evolution.

β-Catenin signalling is essential for gastrulation and oral development in *N. vectensis*^34^,^35^. In this study, we demonstrate that β-catenin is the early inducer of the oral nervous system of *N. vectensis*. β-Catenin signalling is highly activated at the future oral side at the blastula stage and even before (Supplementary Fig. 8)^34^, which causes an upregulation of early and late neurogenic and neural marker genes at the oral side, while inhibition of β-catenin showed opposite effects. Thus, β-catenin signalling is essential for early cell fate decisions of progenitor cells giving rise to multiple neural cell types. Since the target genes of β-catenin signalling, for example, *NvBra* and *NvAshB*, start being upregulated before canonical Wnt ligands *NvWnt3* and *NvWnt4* become detectable^55^, the higher activity of β-catenin signalling at the future oral side of early embryos might include NvDishevelled-mediated stabilization of β-catenin protein at the oral region^56^.

Another intriguing finding revealed in this study is the β-catenin dependency of expression of the early lateral neural genes *NvAshA*, *NvSoxB2c* and *NvElav1* at the blastula stage. Although it is yet unknown how the locally activated β-catenin signalling contributes to the early expression of these genes at the blastula lateral epithelium (for example, we cannot rule out that the effects of β-catenin in neuronal cell formation are indirect), our findings may suggest that β-catenin protein that is expressed uniformly in early blastomeres has a role to predispose the blastula epithelium to become neurogenic, as it has been suggested by previous studies on vertebrates^57^. Our data support observations made in distinct bilaterians showing a function of β-catenin signalling in early neurodevelopment^29^,^57^,^58^,^59^,^60^.

When we tested the function of Bmp signalling on the early induction of oral neuronal genes, we could not detect any inhibitory Bmp effect, which was unexpected since Bmp/Chordin signalling is frequently considered as the primary and general CNS inducer^15^,^20^. Our findings are, however, in accord with data on early neural development in several slow-evolving bilaterians. In annelids^22^ and in hemichordates^24^, Bmp treatments did not inhibit early neurogenic gene expression. We therefore conclude that the ancestral mode of neural induction is probably not the Bmp-mediated event, which was described for *Xenopus* and *Drosophila*^21^. Instead, the β-catenin signalling dependency of neural development at the blastoporal site appears to represent the evolutionary conserved and ancestral mode of neural development with a deep root in the common ancestor of cnidarians and bilaterians.

Treatment of gastrulae and planula larvae with inhibitors of GSK3 (for example, ALP or 1-AZA) triggered β-catenin activation and the expansion of oral markers including RFa^+^ neurons^6^, or even the formation of multiple polyp heads^52^. This clearly suggests an additional function of Wnt/β-catenin signalling for maintaining and/or patterning the cnidarian nervous system. Since the oral end of the cnidarian body plan is considered to represent the posterior end of the bilaterian AP body axis^61^,^62^,^63^, Wnt/β-catenin signalling might also be involved in the oral–aboral patterning of the pervasive cnidarian nerve net. Bmp signalling was also postulated, however, to have a role in maintaining RFa^+^ neurons at the planula stage^36^. How can these observations fit with our principal finding that Bmp signalling is indispensable for development of the oral nervous system in *N. vectensis*? When we analysed Bmp signalling at the planula stage, we discovered that RFa^+^ and GLWa^+^ neurons as well as early and late oral neurogenic TFs (*NvArp2*, *-3*, *-4*, *-5*, *-7*, *NvAshB*, *-C*, -*D*) exhibited indeed a distinct Bmp sensitivity. Only for *NvAshB* this Bmp dependency was weak, and it was absent for *NvSoxB2a*. Therefore, Bmp signalling is an important factor in the development of the oral nervous system. However, this Bmp regulation is complex. When Bmp signalling was either suppressed or over-activated at gastrula and planula stages, neurogenic TFs and GLWamide neuropeptide became downregulated under both conditions. This is reminiscent to the blastoporal regulation of *NvSoxB1* and *NvNk2.1* that are likewise downregulated in *NvChordin* and *NvBmp2/4* morphants^36^. Thus, a balanced level of Bmp signalling seems to be crucial for the development of the oral nervous system. Because the expression of *NvBmp2/4*, *NvBmp5–8* and *NvChordin* genes is dependent on β-catenin signalling, the Wnt/β-catenin regulation of neural patterning might be mediated, at least in part, by the Bmp activity. This point is of interest for future studies.

A striking function of Bmp signalling at the planula stage is the asymmetric induction of neurons along the (secondary) directive axis. *NvArp6*-expressing cells and GLWa^+^ neurons form a distinct asymmetric pattern along the directive axis, which is Bmp dependent. This asymmetric suppression of Bmp is reminiscent to an evolutionarily conserved Bmp function in the mediolateral patterning of the trunk nervous system in bilaterians^22^. It should be pointed out that Bmp inhibition (which activates *NvArp6* but suppresses other oral neuronal TFs) did not result in more and symmetric development of GLWa^+^ neurons. Therefore, GLWa^+^ neurons depend on additional neurogenic TFs, which are similarly restricted to the oral site.

**Figure 9:**
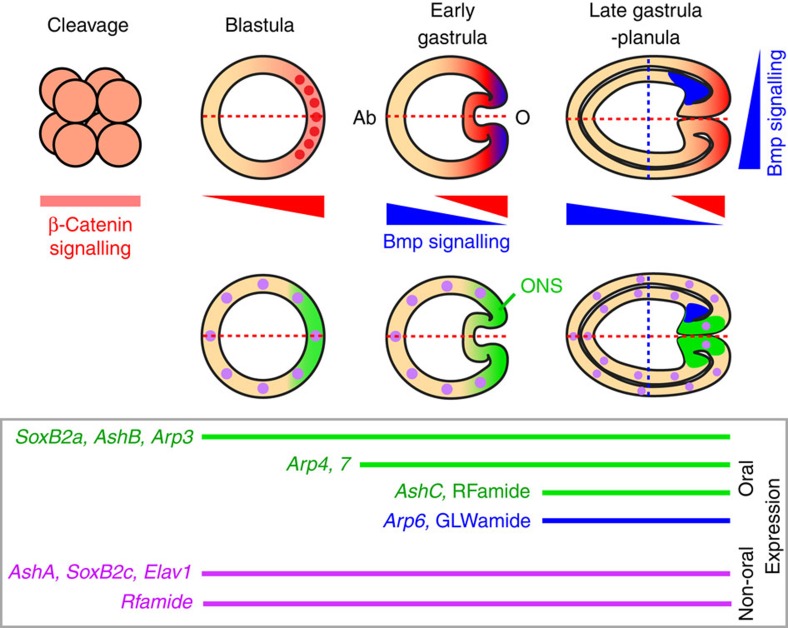
β-Catenin and Bmp regulate early *Nematostella* neurogenesis. The embryonic regions of the gradient of β-catenin activation (red), Bmp activation (blue) and oral nervous system (ONS) (green) in different embryonic stages of *N. vectensis* are shown. β-Catenin protein is observed in all blastomeres at the early-cleavage stage, and stabilized more at the prospective oral side (O) than aboral side (Ab) along the primary axis (red dashed line) of the blastula, which induces the expression of the early neurogenic genes (*NvSoxB2a*, *NvAshB* and *NvArp3*) as well as *NvBmp2/4*, *NvBmp5–8* and *NvChordin* at the oral region. β-Catenin-translocated nuclei at the presumptive blastopore region are shown in red dots. During gastrulation, Bmp signalling starts to be involved in the development of the oral nervous system with respect to the primary and secondary (blue dashed line) body axes. At the planula stage, asymmetric suppression of Bmp activity along the secondary axis becomes indispensable for the expression of *NvArp6* and development of GLWa^+^ neurons at the one side of the oral endoderm.

Our data provide a new view on the evolution of the nervous system, and we propose that the cnidarian oral nervous system exhibits some important features that are essential for the evolution of the bilaterian CNS (Fig. 9). (i) The oral nervous system is demarcated from other parts of the diffused nervous system and exhibits a distinct condensation of neuronal perikaria with neuronal processes extending to the periphery (tentacles and aboral side). The condensations of neuronal perikaria in the oral nervous system is an anatomical feature related to the formation of a CNS in bilaterians with distinct and functionally specialized neuronal domains (ganglia)^64^. (ii) Development of the cnidarian oral nervous system is dependent on early β-catenin- and late Bmp signalling activities. The function of these signalling pathways in the oral nervous system development is highly reminiscent to their function in the specification of neural cell types in the CNS along bilaterian body axes. (iii) There is an evidence for a common set of orthologous neuronal genes expressed in the cnidarian oral nervous system and the bilaterian CNS. The neuropeptides belonging to R[F/Y]amide and [G/V/L]Wamide groups have been shown to form clusters in bilaterian CNS^41^,^42^. In addition to neurogenic TFs used in this study, some of marker genes for bilaterians CNS (that is, *SoxB1* and *Nk2.1)* are exclusively expressed in the oral nervous system in *N. vectensis*^12^,^36^. These findings suggest that the genetic signature of the cnidarian oral nervous system is shared, at least in part, by that of the bilaterian CNS.

Most recently the genome of comb jellies (ctenophores), another pre-bilaterian clade with a primitive nervous system, was published^65^,^66^. Many neuronal genes are missing in ctenophores, for example, those for the synthesis and transmission of the classical neurotransmitters GABA and acetylcholine. However, several of the ‘neurogenic’ TFs (for example, SoxB and bHLH) and RNA-binding proteins (Elav and Musashi) are present in ctenophore genomes^65^,^66^. In the ctenophore, SoxB and RFamide are strongly expressed at neuron-enriched body regions, that is, the aboral sensory organ and the polar fields^67^,^68^. In addition, members of Bmp and β-catenin signalling are also highly expressed at this region during development^69^,^70^. The fact that these markers are expressed during the neural development in ctenophores is questioning, whether the nervous system of ctenophores had really a different evolutionary origin from the cnidarian-bilaterian clade as recently proposed^66^.

To understand the emergence of the bilaterian CNS from simple neural clusters, deeper insights into the anatomical and molecular features of the cnidarian nervous systems are very helpful. Although this knowledge is still far from being complete, our data on the development of the oral nervous system in *N. vectensis* give an insight how a first step in the evolution of nervous system centralization may have been accomplished. The cnidarian nervous system is also an intriguing example, how basic positional information, which is organized in bilaterians in a Cartesian coordinate system^26^, can be translated into the development of the nervous system.

## Methods

### Culture of *N. vectensis*

*N. vectensis* were cultured at a salinity one-third of normal seawater (that is, brackish water, 108.32 g l^−1^ Tropic Marine sea salt, pH 7.6). Adult males and females were separately cultured at 18 °C in the dark and fed two to three times per week. On 2 days after the feeding, the culture boxes were washed. For spawning the release of eggs and sperms, the culture boxes were incubated at 26 °C under the light for at least 14 h. After fertilization, embryos were cultured at 20 °C.

### Gene isolation from *N. vectensis* and plasmid construction

Gene-specific primers were then designed to recover both 3′ and 5′ rapid amplification of complementary DNA (cDNA) ends (RACE) fragments using the GeneRacer kit (Invitrogen) with annealing temperatures between 55 and 65 °C for 1st and nested PCR. Gene-specific primer sequences are listed in Supplementary Table 1. RACE products were cloned using pGEM-T (Promega) and sequenced. Overlapping 5′and 3′ RACE fragments were aligned to obtain mRNA sequences. The DN form of GSK3β (K83R) was made by two-step PCR-based mutagenesis. The PCR product was inserted into the pCS2 plasmid and sequenced. For the TnT assays, *NvSoxB2a*, *NvAshB*, *NvArp3* and *NvArp6* genes were subcloned with partial 5′ untranslated region (UTR) and the complete open-reading frame into pGEM-T vectors and sequenced. The insert was cut using EcoRI and inserted into the pCS2 plasmid.

### Quantitative and semi-quantitative RT-PCR

For semi-quantitative RT-PCR, the level of amplified product was compared in the exponential range of amplification. qRT-PCR was performed on the Bio-Rad DNAEngine equipped with Chromo4 real-time PCR detector (Bio-Rad) using Absolute QPCR SYBR Green mix (Thermo Scientific). The primer sequences are listed in Supplementary Table 1.

### Embryo treatment with Bmp protein or GSK3β inhibitors

Embryos were treated with recombinant hBmp2 (a gift from Dr Wölfl) at concentrations from 0.1 to 1 μg ml^−1^. As a control, 1 μg ml^−1^ bovine serum albumin (BSA) was applied. hBmp2 protein was added at one-two-cell-stage embryos and cDNAs were prepared at the blastula (12–14 h.p.f.), gastrula (30 h.p.f.) or planula (74 h.p.f.) stages. For WISH experiments, hBmp-treated blastulae and gastrulae (12 h.p.f. (blastula) and 24 h.p.f. (gastrula) were fixed. For experiments using the GSK3β inhibitors to enhance β-catenin signalling, embryos were treated with ALP, BIO or 1-AZA (Sigma) at the concentrations indicated. cDNAs were prepared at the blastula and early gastrula stages (12–18 h.p.f.) for the qPCR analyses. For immunostaining of neuropeptides and mOrange protein, embryos were treated with GSK3β inhibitors from one- and two-cell stage to blastula stage. After being washed, the embryos were cultured in *Nematostella* medium until early (72 h.p.f.) or late (98 h.p.f.) planula stages. To pharmacologically inhibit β-catenin signalling, embryos were treated with 10 μM or 50 μM iCRT14 (Sigma), a Tcf/β-catenin inhibitor, for 14 h (blastula), 3 days (early planula) or 4 days (late planula).

### Microinjection of mRNA and MO antisense oligonucleotides

pCS2 constructs, pCS2-MT-β-Eng (DN-β-catenin) (a gift from Dr McCrea), pCS2-NvBmp2/4 and DN-GSK3β, were linearized using appropriate restriction enzymes. Capped mRNAs were synthesized using the SP6 mMessage mMachine mRNA synthesis kit (Ambion). Capped mRNAs or MO antisense oligonucleotide for control (5′- CCTCTTACCTCAGTTACAATTTATA ), neurogenic TFs NvSoxB2a (MO1: 5′- TCTAAATCCCGTAGAAGTTCTAGGT -3′, MO2: 5′- CGTCAAGTTTACTCGTTCCGGCACT -3′), NvAshB (MO1: 5′- AGGAAGCCTCCATCGGAATCTCCAT -3′, MO2: 5′- TTGTAAGCGAGAAGCACTCACATGC -3′), NvArp3 (MO1: 5′- TTTCTGTACCTTCGCCTTTTGTCAT -3′, MO2: 5′- CTGAGAGCCGGTGAGTTTTCTTGAT -3′), NvArp6 (MO1: 5′- TCATTGAGTGAGTGCATCCGGCTTC -3′, MO2: 5′- GCGCACTCTCTGTTTGATCGTAAGT -3′), NvBmp2/4 (5′- GTAAGAAACAGCGTAAGAGAAGCAT -3′)^36^, NvBmp5–8 (5′- GTAACAGGTCTCGTATTCTCCGCAT -3′)^36^ and NvTcf (5′- CTGAGGCATACTACCGTCTCATGTG -3′)^55^ were injected at 250 μM concentration into fertilized eggs with 0.2 μg μl^−1^ rhodamine- or Alxa488-dextran (Invitrogen). Embryos were then rinsed with *N. vectensis* medium and incubated until desired stages.

### TnT assay

The translation-blocking MOs were tested using the TnT SP6 quick coupled transcription/translation system (Promega). pCS2 plasmids (250–500 ng) containing MO-target sites (5′ UTR and first ATG) were incubated with control or gene-specific MOs (50 μM) at 30 °C for 90 min. The biotinylated proteins synthesized were detected by western blotting using horseradish peroxidase-conjugated streptavidin (1:10,000 dilution).

### Whole-mount *in situ* hybridization

*In situ* hybridization was performed as described previously^37^ with following modifications: specimens were fixed with 4% paraformaldehyde/PBST (PBS, 0.1% Tween 20) for 1 h, then washed with methanol for 3 times and stored at −20 °C. Hybridization of 0.6- to 1.4-kb digoxygenin-labelled antisense RNA probes were carried out using a hybridization solution containing 1% SDS at 50–65 °C for at least 22 h. For post-hybridization washes, specimens were washed by serial dilutions (75, 50 and 25%) of hybridization solution with 2 × SSC at 55 °C. After processing of the digoxygenin-labelled probe with BM purple (Roche), specimens were washed with PBST.

### Western blotting

*N. vectensis* embryos were solubilized with 2 × SDS–polyacrylamide gel electrophoresis sample buffer and immediately boiled at 90 °C for 5 min. After being sonicated, proteins separated by SDS–polyacrylamide gel electrophoresis were transferred to a PVDF (polyvinylidene difluoride) membrane (Millipore). The membranes were blocked with 5% BSA in buffer consisting of 50 mM Tris-HCl, pH 8.0, 2 mM CaCl_2_, 80 mM NaCl and 0.2% Nonidet P-40, incubated with the blocking solution containing anti-phospho-Smad1/5/8 antibody (Cell Signaling Technology) (1:200 dilution), anti-Smad1/5/8 (N-18) antibody (Santa Cruz Biotechnology) (1:300 dilution) or anti-β-tubulin (2-28-33) antibody (Sigma) (1:1,000 dilution) followed by incubation with horseradish peroxidase-conjugated secondary antibodies (Jackson ImmunoResearch) (1:5,000 dilution) in blocking solution. Immunoreactive proteins were detected with an ECL immunoblotting detection reagent (Amersham Pharmacia Biotech). Full images of western blots are shown in Supplementary Fig. 15.

### Immunostaining and fluorescent microscopy

The NvElav1::mOrange transgenic embryos were fixed with 4% paraformaldehyde/PBT for 1 h at room temperature and stained using anti-DsRed antibody (1:300 dilution)(Clontech), as was previously reported^14^. For neuropeptide staining, embryos and primary polyps were fixed by Zamboni’s fixative o/n at 4 °C. After permeabilization in PBS+0.1% Triton X-100, specimens were blocked with blocking solution (10 mg ml^−1^ BSA+5% normal goat serum in PBS), incubated with anti-FMRFamide antibody (1:600 dilution) (Sigma) or anti-GLWamide antibody (1:400 dilution) (a gift from Dr Koizumi) in the blocking solution containing 0.1% Triton X-100, and then with Alexa488- or Alexa568-conjugated anti-Rabbit antibody (1:800 dilution) (Invitrogen). Specimens were imaged using a Nikon ECLIPSE 80i fluorescent microscope equipped with DIGITAL SIGH DSU1 (Nikon). For confocal microscopy, a fluorescent microscope ECLIPSE Ti (Nikon) equipped with an A1R MP confocal scanner (Nikon) was used.

## Author contributions

H.W. performed most of the experiments. A.K. and M.F. performed some WISH analyses. H.W., K.A., S.Ö., T.F. and T.W.H. designed the project. H.W. and T.W.H. wrote the manuscript.

## Additional information

**How to cite this article:** Watanabe, H. *et al*. Sequential actions of β-catenin and Bmp pattern the oral nerve net in *Nematostella vectensis*. *Nat. Commun.* 5:5536 doi: 10.1038/ncomms6536 (2014).

## Supplementary Material

Supplementary Information Supplementary Figures 1-15 and Supplementary Table 1
