# Supplementary material for: Sequential actions of β-catenin and Bmp pattern the oral nerve net in Nematostella vectensis
Source: Nat Commun. 2014 Dec 23;5:5536. doi: 10.1038/ncomms6536 (PMC4284808; doi:10.1038/ncomms6536)
Supplement: Supplementary Information — Supplementary Figures 1-15 and Supplementary Table 1 [file ncomms6536-s1.pdf]

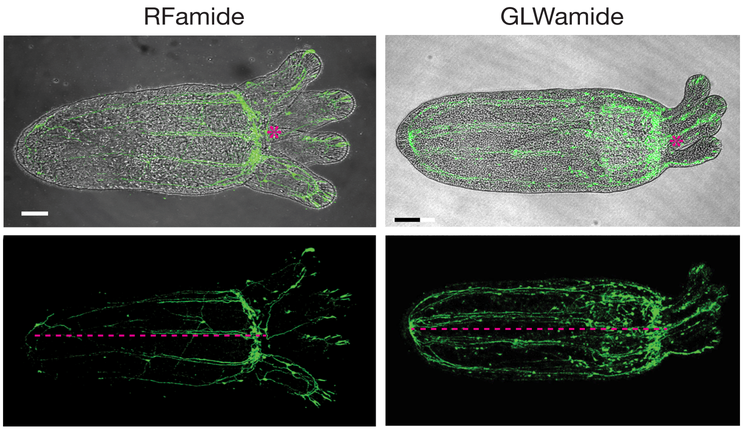

Supplementary Figure 1 | RFa+ and GLWa+ neurons around the mouth of *N. vectensis* primary polyps. RFa+ and GLWa+ neurons established a neural plexus connecting the oral and aboral end at the primary polyp stage. Asymmetric organisation of GLWa+ neurons along the directive axis around the pharynx in planula larvae was lost in primary polyps. Shown are representative images of three experiments with similar results. The pink dotted lines denote the oral-aboral axis. Red asterisks indicate the blastopore. Scale bars, 100 μm.

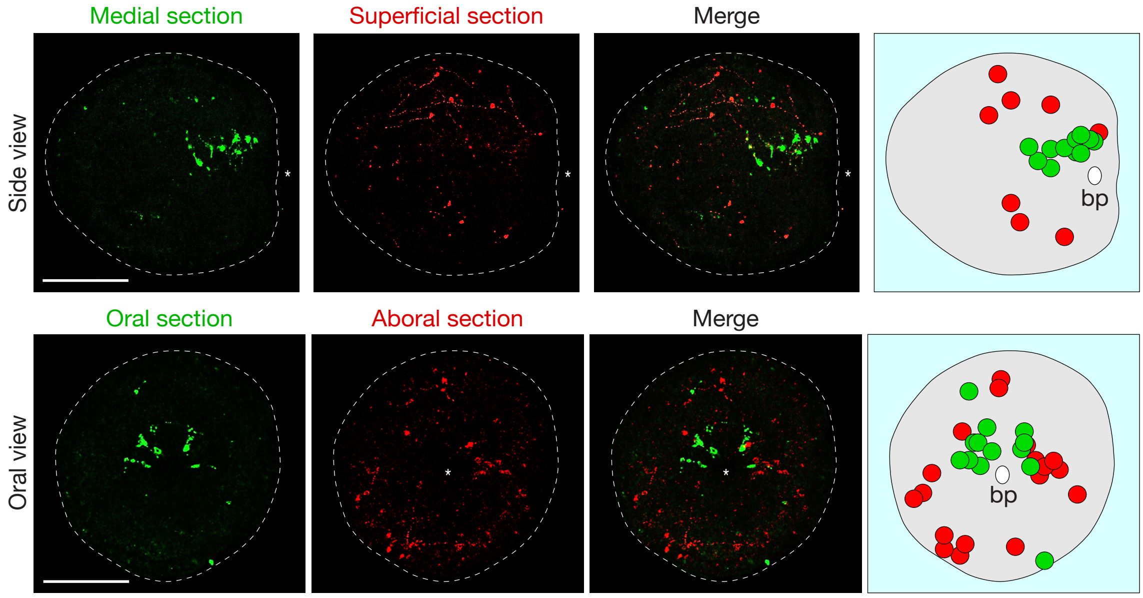

Supplementary Figure 2 | Ectodermal and endodermal GLWa+ neurons in *N. vectensis* planula. Confocal microscopy showed that the GLWa+ neurons formed a cluster at one side of the pharyngeal endoderm (medial and oral sections, shown in green), whereas ectodermal GLWa+ neurons were distributed rather evenly along the directive axis (superficial and aboral sections, shown in red). Shown are representative images of three experiments with similar results.

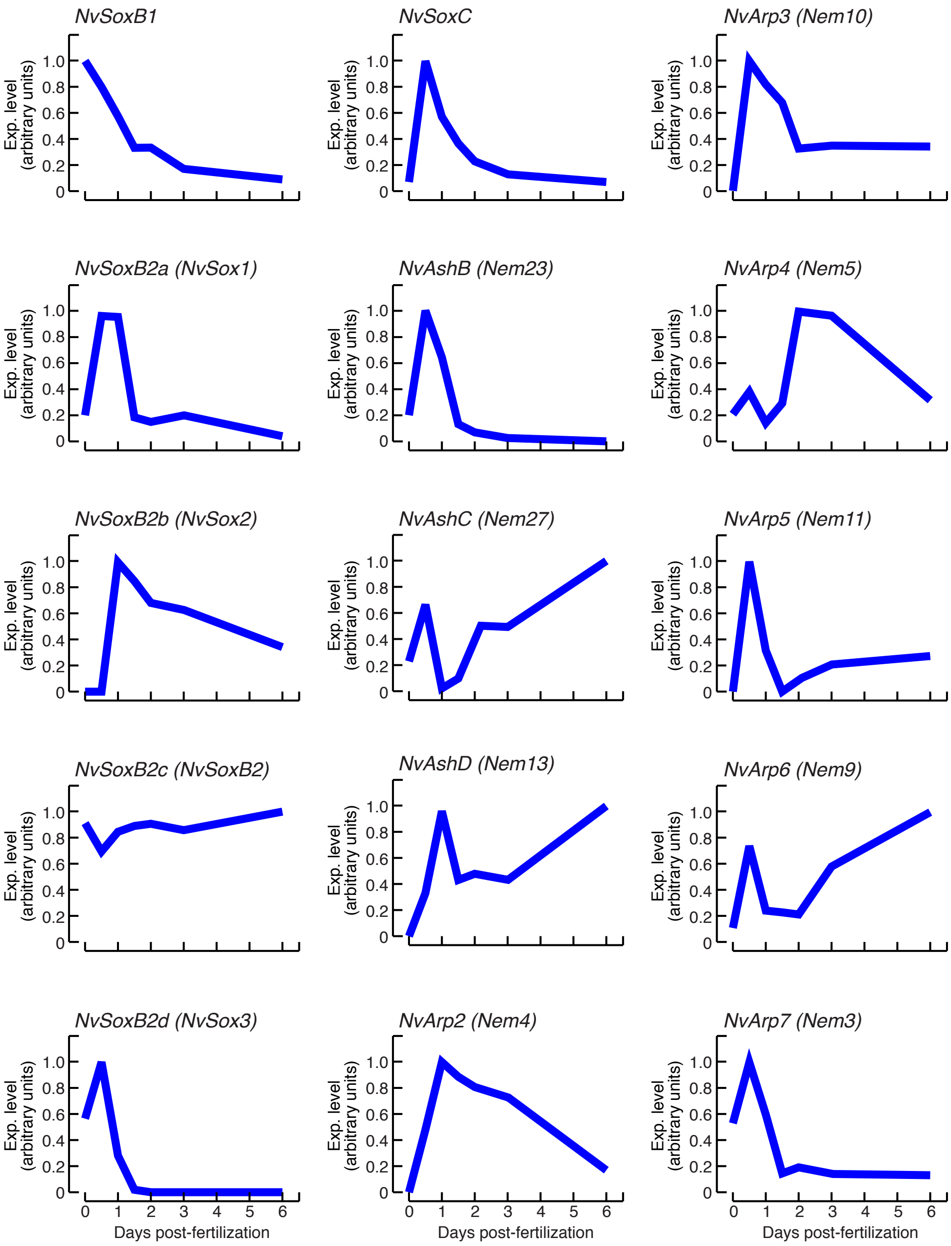

Supplementary Figure 3 | Semi-quantitative analysis of neurogenic genes in N. vectensis embryos. *NvSoxB1* was maternally and abundantly expressed in the unfertilized egg and down-regulated after gastrulation started, *NvSoxB2a* and *NvSoxB2d* were transiently up-regulated at blastula stage. Several orthologous proneural genes, especially *NvAshB* and *NvArp3* started to be up-regulated at the blastula stage. Although up-regulation of *NvAshC*, *NvAshD*, *NvArp2*, *NvArp4*, and *NvArp5* was observed during early embryogenesis, these neural bHLH transcription factors were evidently expressed on later developmental stages, in the planula larva and primary polyp, suggesting their multiple roles for neuroblast and later neural cell type specification. *NvEf1α* was used to normalize the samples. Shown are representative images of three experiments with similar results.

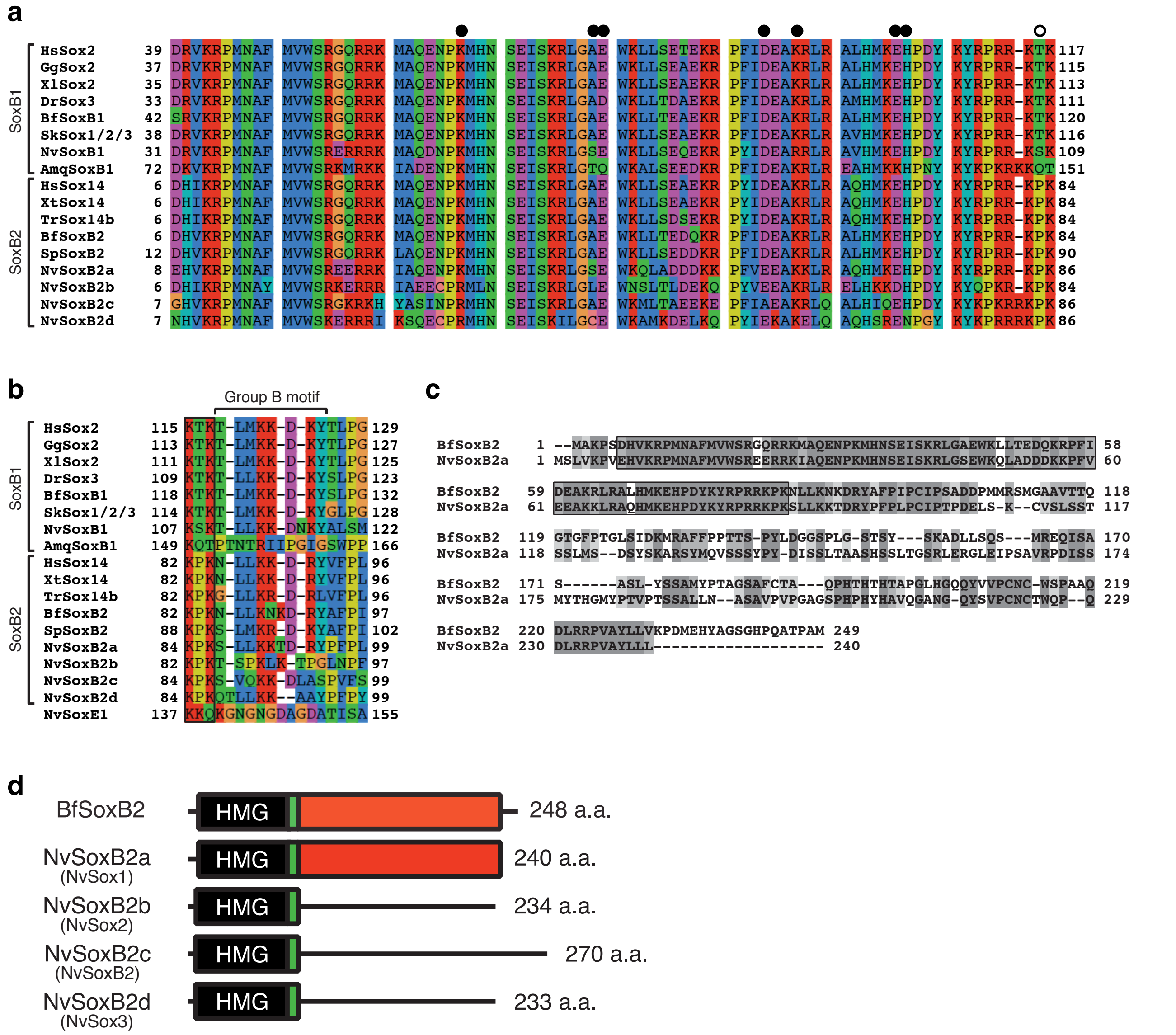

Supplementary Figure 4 | Comparisons of the domain organization of SoxB proteins. (**a**) Alignment of amino acid sequences of SoxB HMG domains of bilaterians, sponge and sea anemone N. vectensis. The characteristic amino acids for group B (filled circles) and subgroups B1/B2 (open circles) are indicated. The HMG domains of these *N. vectensis* Sox genes also show identity/similarity on the amino acid level characteristic of group B. (**b**) A comparison of group B motifs. Note that the highest similarity to group B specific motifs of the bilaterian SoxB2 was observed in NvSoxB2a. The HMG domains are boxed. (**c**) A comparison of full-length amino acid sequences of SoxB2 proteins of *Branchiostoma floridae* (BfSoxB2) and *N. vectensis* (NvSoxB2a). Identical and similar amino acid residues are shaded heavily and lightly, respectively. The HMG domains are boxed. (**d**) Scheme of the *B. floridae* and *N. vectensis* group B2 Sox proteins. Numbers at the ends of the bars represent the total number of amino acids. Conserved Group B specific amino acids are shown in green boxes. C-terminal domains that bear evident conservation with chordate SoxB2 subgroup are shown in red boxes. The *N. vectensis* gene names that have been previously defined are shown in the parentheses. Hs, *Homo sapiens*; Gg, *Gallus gallus*; Xl, *Xenopus laevis*; Dr, *Danio rerio*; Bf, *Branchiostoma floridae*; Sk, *Saccoglossus kowalevskii*; Nv, *Nematostella vectensis*; Amq, *Amphimedon queenslandica*; Xt, *Xenopus tropicalis*; Tr, *Takifugu rubripes*; Sp, *Strongylocentrotus purpuratus.*

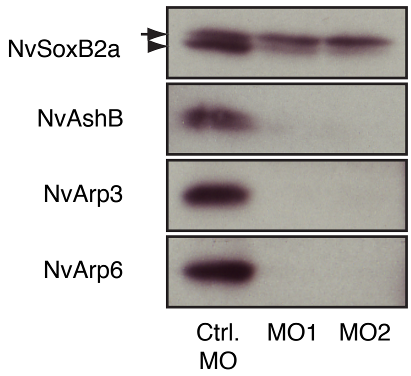

Supplementary Figure 5 | Efficiencies of the translation-blocking morpholinos against *NvSoxB2a*, *NvAshB*, *NvArp3* and *NvArp6*. The pCS2 plasmids containing partial 5' UTR and the complete open reading frame of the *NvSoxB2a*, *NvAshB*, *NvArp3* or *NvArp6* were incubated with corresponding morpholinos (50 μM). The Arrow on NvSoxB2a indicates a non-specific protein product. NvSoxB2a protein is denoted with the arrowhead. Shown are representative images of three experiments with similar results.

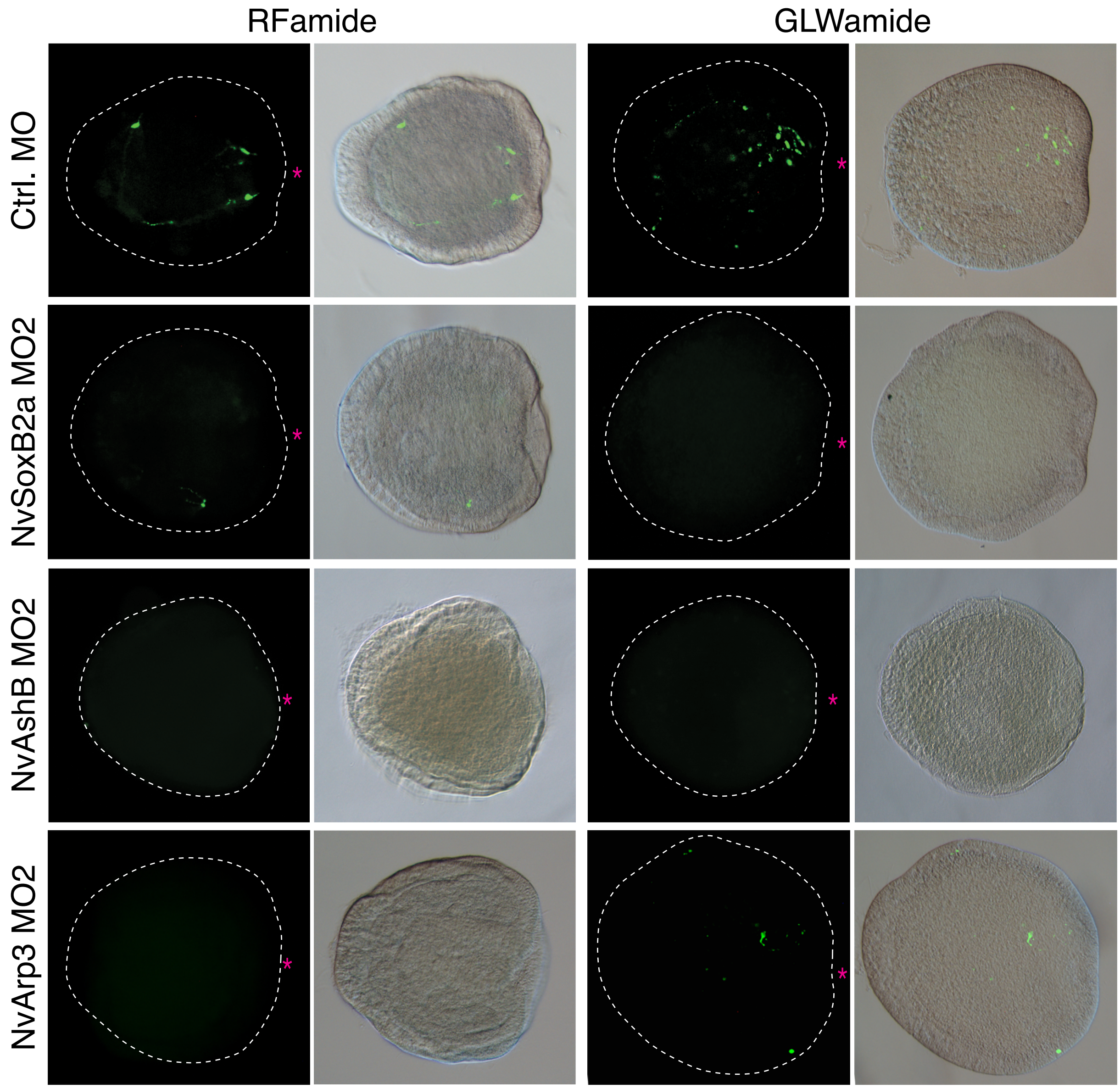

Supplementary Figure 6 | Early neurogenic gene functions are required for neural development. Immunohistochemistry using anti-RFa and anti-GLWa neuropeptides revealed that development of the RFa+ and GLWa+ neurons in the planula larvae was strongly inhibited by injection of morpholinos against *NvSoxB2a*, *NvAshB* and *NvArp3* transcription factors. Shown are representative images of three experiments with similar results. The pink asterisks indicate the blastopore.

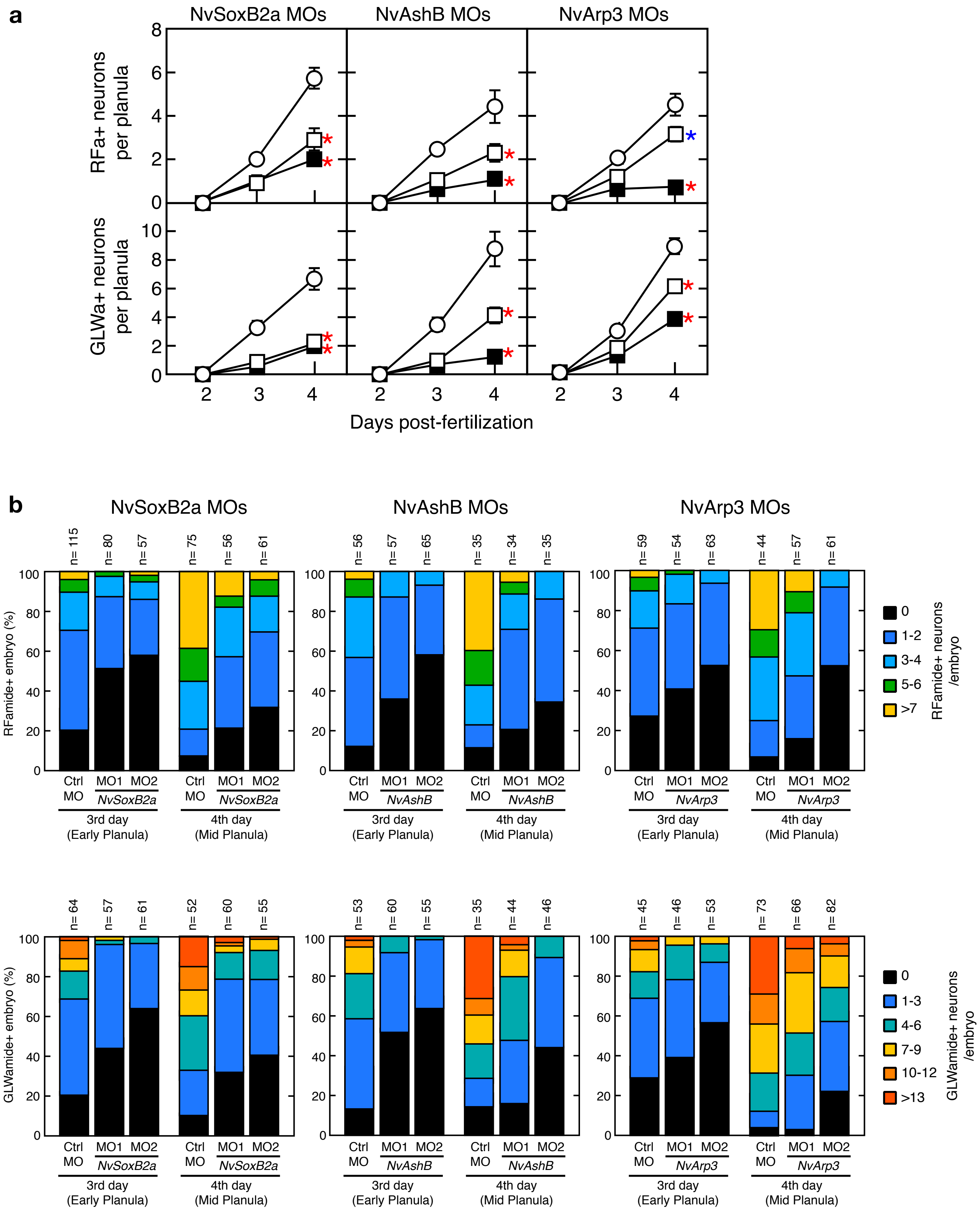

Supplementary Figure 7 | RFa+ and GLWa+ neurons in *NvSoxB2a*, *NvAshB* and *NvArp3* morphants. Quantification of number of RFa+ and GLWa+ neurons at the planula larvae that was injected morpholinos against *NvSoxB2a*, *NvAshB* or *NvArp3*. (**a**) The number of RFa+ and GLWa+ neurons was decreased in planula larvae injected with morpholinos against the early neurogenic genes. The data represent the mean ± s. e. m. of at least three experiments. Asterisks denote statistical significance with student's t-test (*, p< 0.01). (**b**) Numbers of larvae with RFa+ and GLWa+ neurons were also decreased in the morphants.

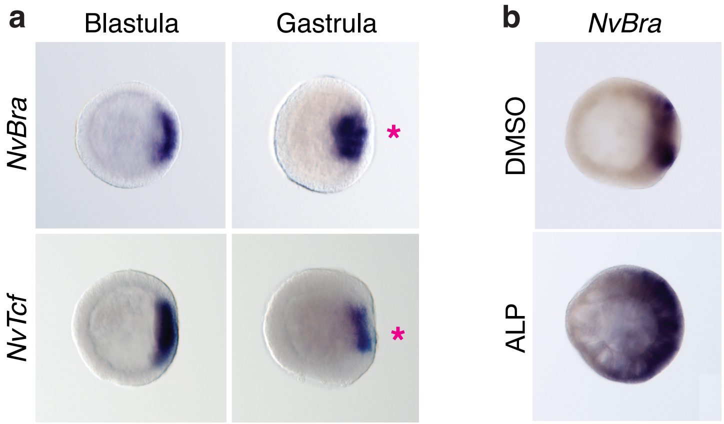

Supplementary Figure 8 | Expression of *NvBrachyury* and *NvTcf* at the prospective blastopore. (**a**) WISH analysis demonstrated that the β-Catenin target genes *NvBrachyury* (*NvBra*) and *NvTcf* start to exclusively be expressed at the prospective blastopore region at blastula stage, indicating a localized activation of β-Catenin signalling at this embryonic region. (**b**) Treatment of early embryos (egg-blastula) with a GSK3β inhibitor ALP (2.5 μM) induced ectopic and increased expression of *NvBra*. Shown are representative images of three experiments with similar results. The pink asterisks indicate the blastopore.

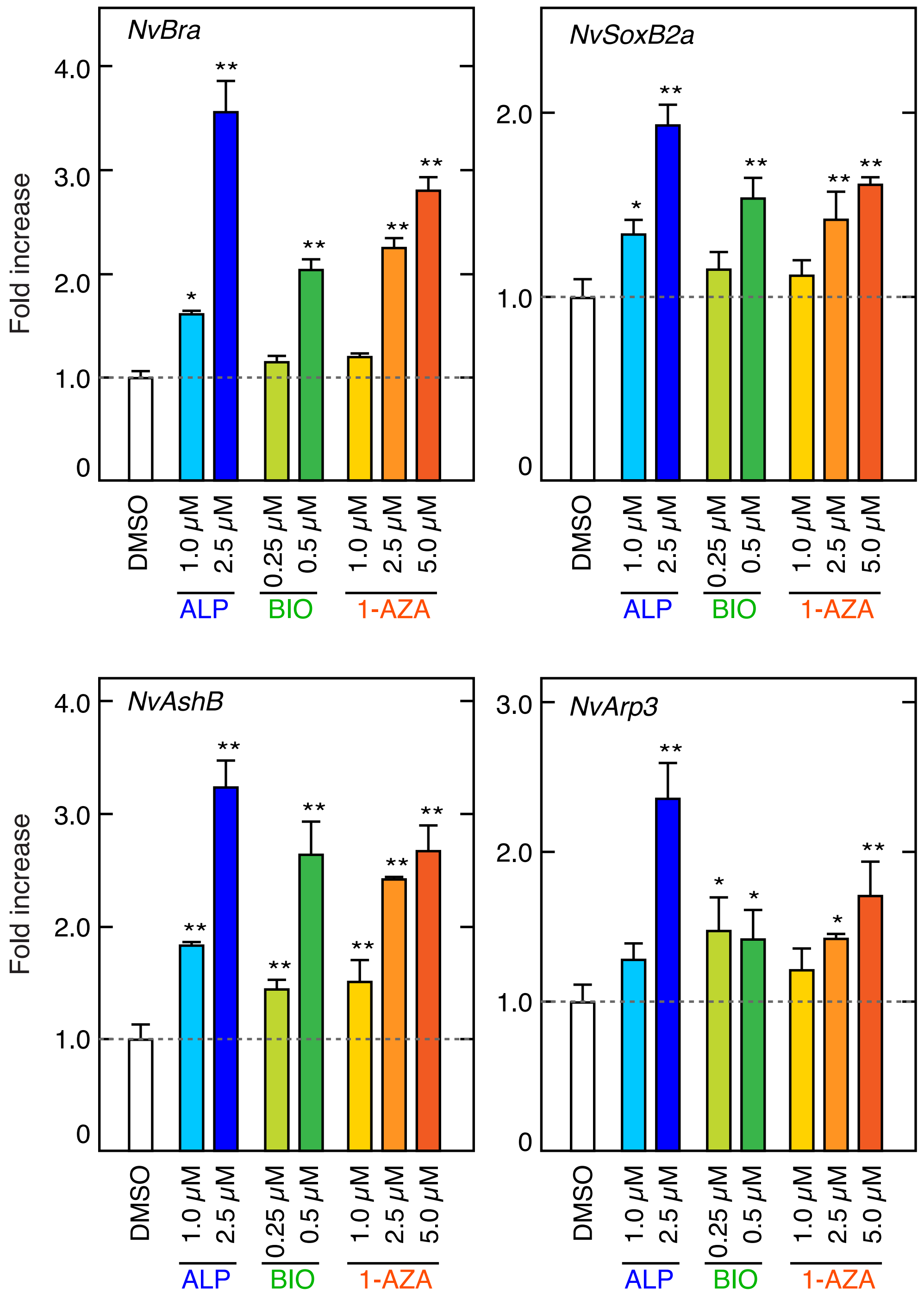

Supplementary Figure 9 | Dose-dependent up-regulation of NvBra and early neurogenic genes by GSK3β inhibitors. qPCR analysis of *NvBra* and neurogenic genes after treatment with increasing concentrations of GSK3β inhibitors, ALP, BIO and 1-AZA. NvEf1α was used as an internal control. Bars represent the mean ± s. e. m. of three experiments. Asterisks denote statistical significance with student's t-test (*, p< 0.05; **, p< 0.01).

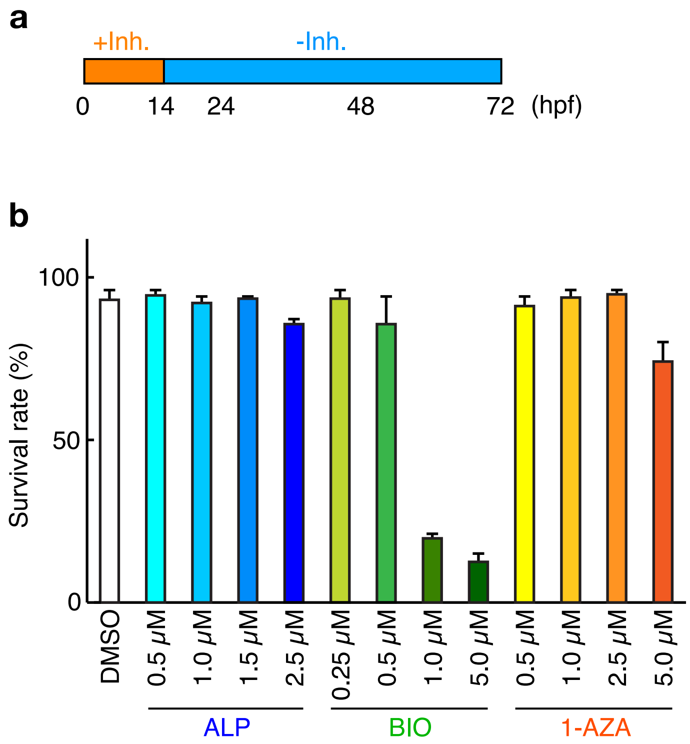

Supplementary Figure 10 | Survival rate of embryos after GSK3β inhibitor treatment. (**a**) A schematic diagram showing the temporal and early treatment with inhibitors. (**b**) Quantification of living planula larvae (72 hpf) treated with indicated concentrations of the GSK3β inhibitors. Bars represent the mean ± s. e. m. of three experiments.

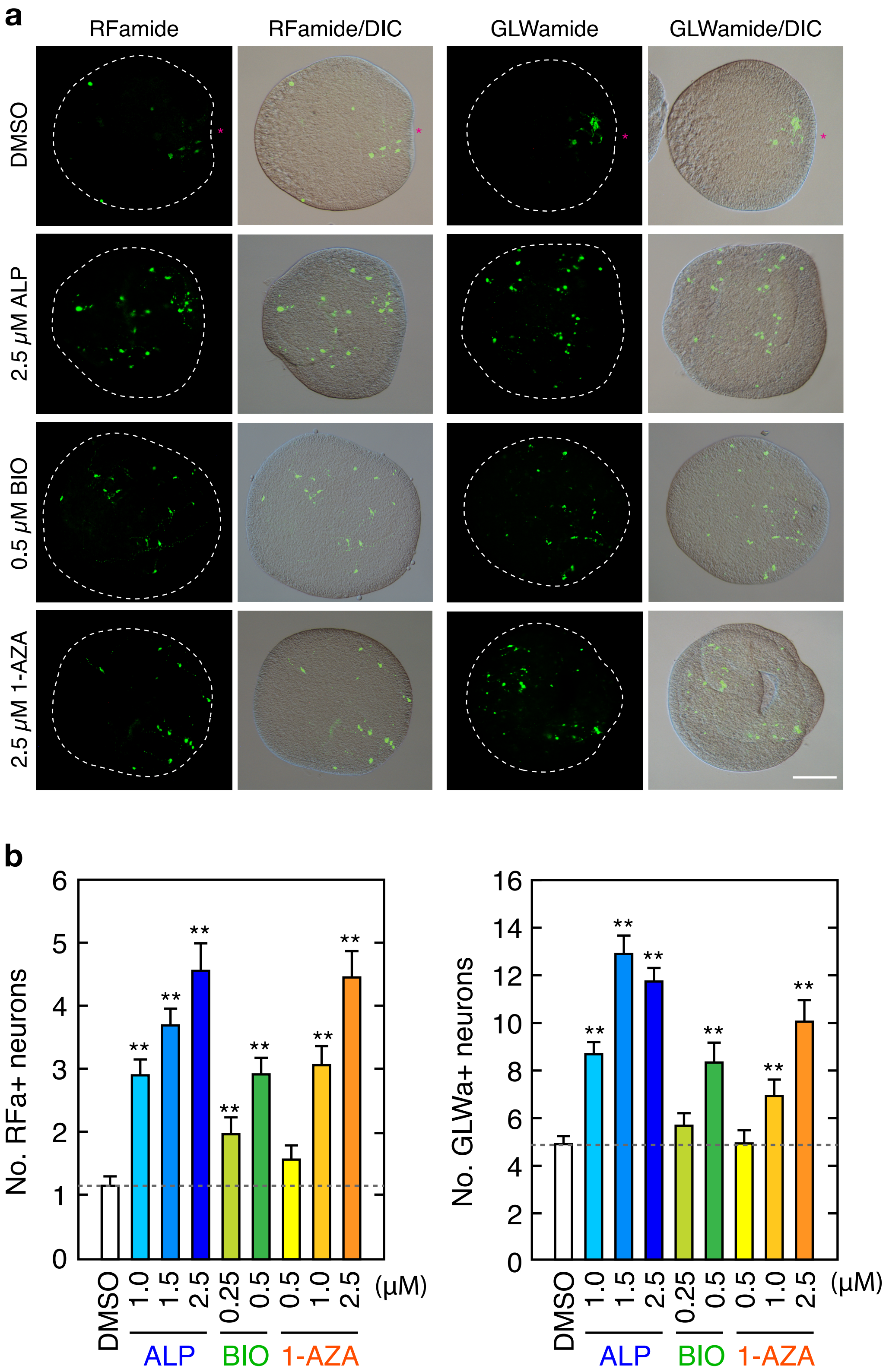

Supplementary Figure 11 | Dose-dependent effect of GSK3β inhibitors on RFa+ and GLWa+ neurons. (**a**) Immunohistochemistry using anti-RFa and anti-GLWa antibodies demonstrated that oral development of these neuropeptide-expressing neurons was expanded toward aboral side in the planula larvae treated with GSK3β inhibitors. The pink asterisks indicate the blastopore. Scale bars, 100 μm. (**b**) The number of RFa+ and GLWa+ neurons was increased in planula larvae treated with GSK3β inhibitors in a dose-dependent manner. Bars represent the mean ± s. e. m. of three experiments. The black asterisks denote statistical significance with student's t-test (**, p< 0.01).

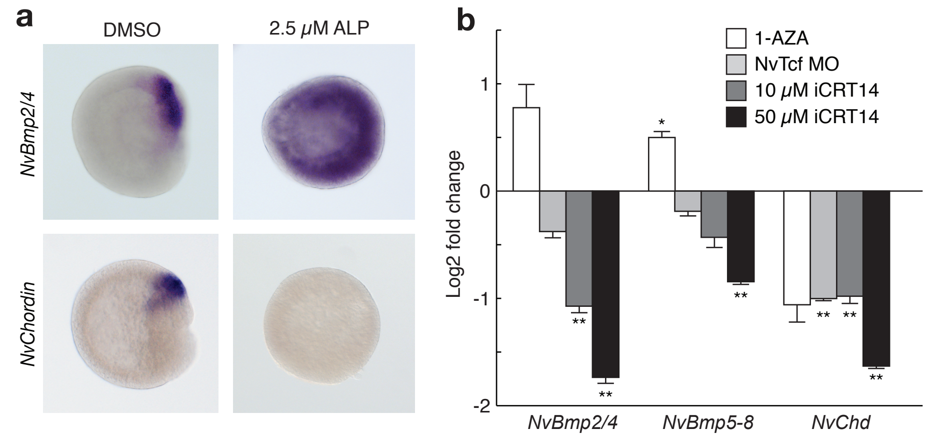

Supplementary Figure 12 | β-Catenin dependent expression of NvBmp2/4 and NvChordin. (**a**) *NvBmp2/4* and *NvChordin* are expressed at one side of the blastopore in gastrula stage. 2.5 μM ALP-treated gastrula showed expanded expression of *NvBmp2/4* in the entire embryos, whereas *NvChordin* turned to being suppressed. (**b**) qPCR analysis of *NvBmp2/4*, *NvBmp5-8* and *NvChordin* at the early gastrula treated with 1-AZA (2.5 μM) or iCRT14 (10 μM and 50 μM), or injected with *NvTcf* morpholino. Note that *NvBmp2/4*, *NvBmp5-8* and *NvChordin* expression required the β-Catenin signalling. The *NvChordin* suppression caused by ALP and 1-AZA was probably due to the dual function of β-Catenin signalling on the *NvChordin* expression. Bars represent the mean ± s. e. m. of three experiments. The black asterisks denote statistical significance with student's t-test (*, $p < 0.05$, **, $p < 0.01$).

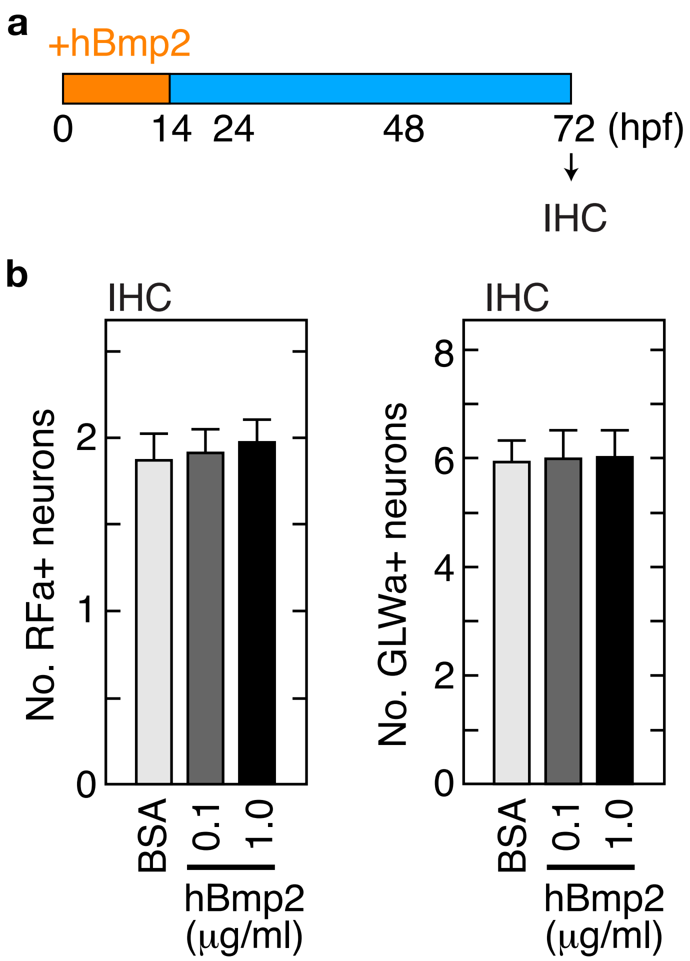

Supplementary Figure 13 | Neurogenesis after over-activation of Bmp signalling. (**a**) A schematic drawing of the temporal hBmp2 treatment during early embryogenesis (egg-blastula). (**b**) Immunohistochemistry using anti-RFa and anti-GLWa antibodies showed that the transient hBmp2 treatment of early embryos does not affect the number of the neuropeptide-expressing neurons at the planula stage. Bars represent the mean ± s. e. m. of at least three experiments.

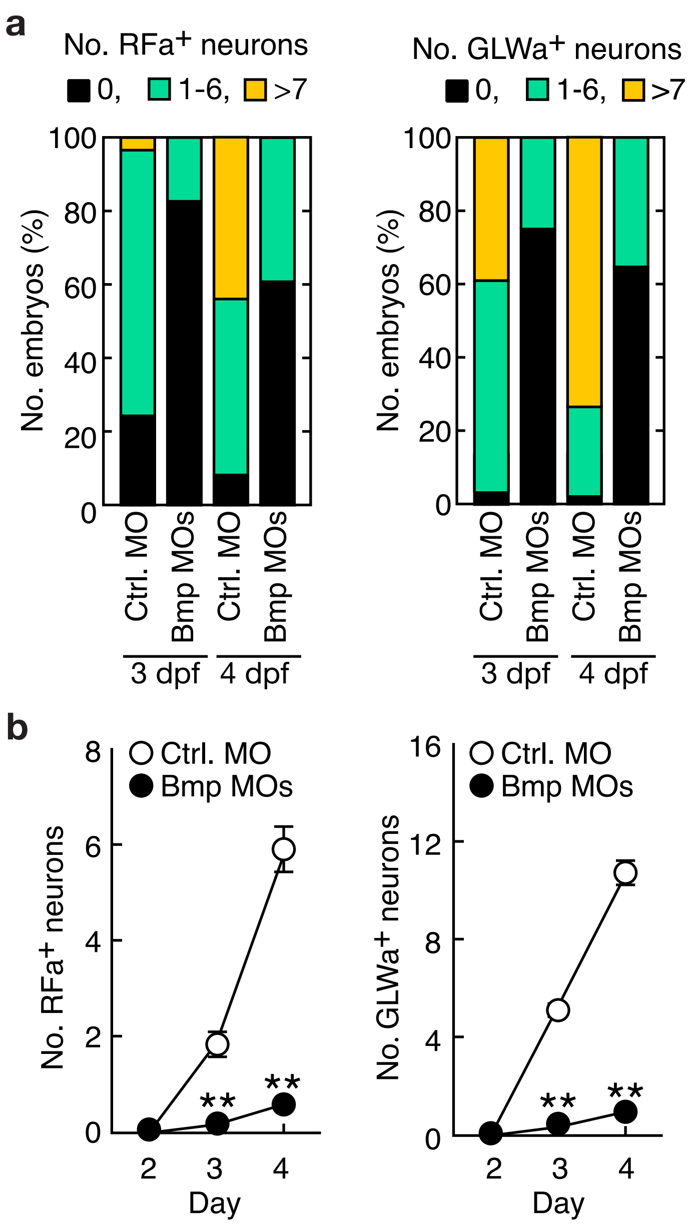

Supplementary Figure 14 | Bmp dependent formation of RFa+ and GLWa+ neurons. (**a,b**) Development of RFa+ and GLWa+ neurons were analysed in the planula larvae injected with morpholinos against NvBmp2/4 and NvBmp5-8. Immunohistochemistry using anti-RFa and anti-GLWa antibodies revealed that the development of these neuropeptide-expressing neurons is depending on the Bmp signalling. The number of larvae with RFa+ and GLWa+ neurons (**a**) and the number of RFa+ and GLWa+ neurons per planula (**b**) were decreased in the *NvBmp2/4/NvBmp5-8* double morphants. The data in (b) represent the mean ± s. e. m. of three experiments. Asterisks in (**b**) denote statistical significance with student's t-test (**, p< 0.01).

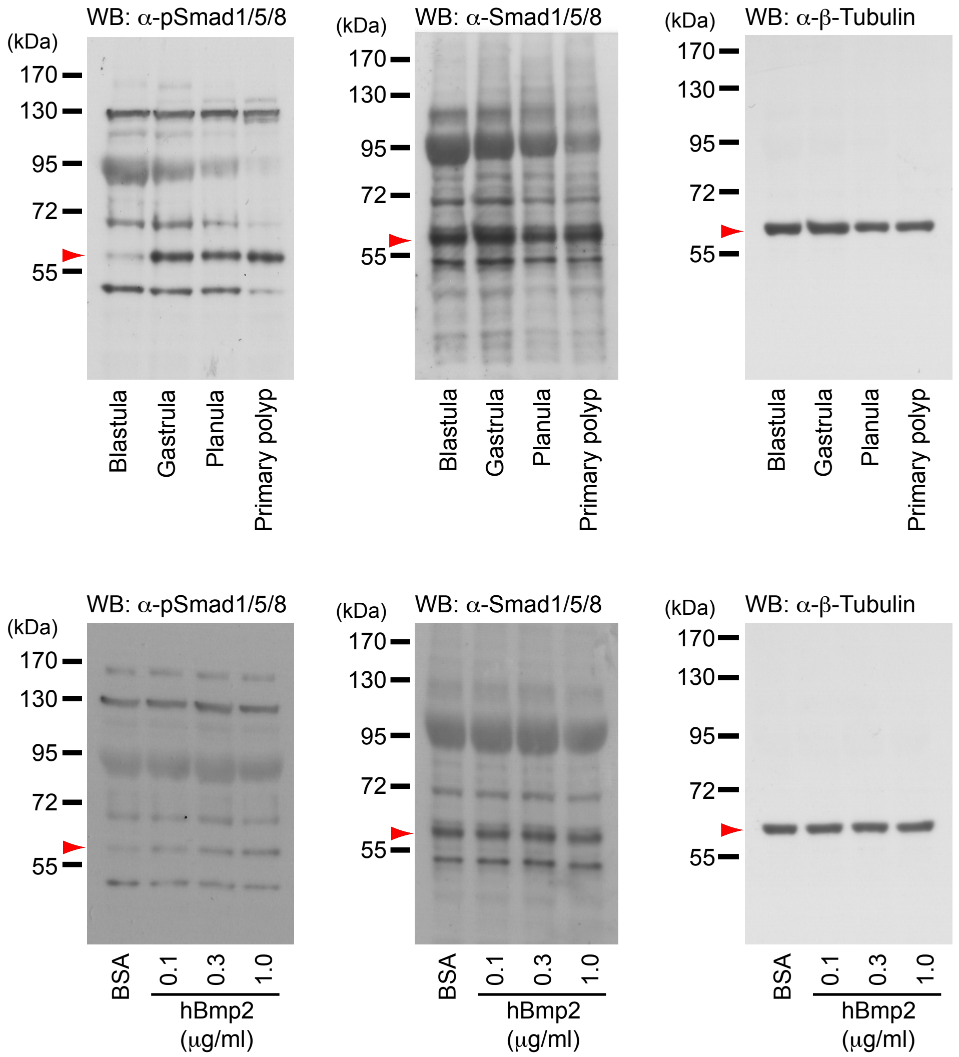

Supplementary Figure 15 | Full images for the Westernblotting (pSmad). Westernblotting analyses were carried out as described in the Method section. Immunoreactive proteins (pSmad1, Smad and β-Tubulin) in developing and hBmp2-treated embryos were detected using ECL reagent with 40 min, 20 min and 10 min exposure time, respectively. Shown are representative images of three experiments with similar results.

Supplementary Table 1: Sequences of primers used for RACE PCR and quantitative PCR analysis

A. Primer sequences for RACE PCR (5'-3')

Gene name	Forward 1st	Forward nested	Reverse 1st	Reverse nested
NvAshB	AGCAGTTCTCAACGTCTCAACT	AGGCGGCGAAGCCGTCACTAA	GGCTTCGCCGCCTTGCGGAAACACA	TGTCCAAAGTTGAGACGTTGAGAA
NvAshC	TCGCAACTTGAATCCGACTTGTT	AGCGGCTCGCAATGTCTCTACT	ATGGCCCAGTACTCACCGTA	TCACCCTATGTACGACTACAATCT
NvAshD	TGCTACCGCGCACCGCAACGTCT	ACGGAATGTGTTGGCGTAGTGAGGA	TGACGGATTTTCCTCACTACGCCAA	GAGACGTTGCGGTGCGCGGTA
NvArp1	CCTCAGTGTGTCCGATAAGGAT	ACTCTGTGGCACAGGGATGCAT	TGCATCCCTGTGCCACAGAGTA	AGAGATTCTCAGGATTGCATGCAAT
NvArp2	TCTCGAGCCAGCAAGAGTGTGTCT	AGCGCCTCTAGCGCTACATTGAT	TGTAGCGCTAGAGGCGCTAAGA	GCTGGCTCGAGACTACATCA
NvArp3	TGGCAGCAGCTTGCGATCCTAT	TCGCCAATCGTAGCACTTCAATCT	TCGCTTGCAGCAGCGGTGAAT	
NvArp4	ATCGCCTTACGGTCGGTGGAACT	TCCGTCGCTTGTTCTGTGAATAT	TGATGTATTACGAGGCGTAATTCCA	TGAGACATTAAGGCTGGCTACACA
NvArp5	TGTCGTGCCTGTAGCAATGTT	TCCCGGAACAACGCTCTTTA	ACGTCCACGACTCAAACTACA	TGCACGCTCAACTCGACAATTGA
NvArp6	TGGTGAACCCTCAGACTCGTT	TCGGTTCAGGCTCAGGTATAGGT	ACTAGCGGCCTTAGAGGAAGA	ACAGAGAGTGCGCAACGGCAA
NvArp7	TCCGCAAGAGCCGATATGTAGCT	TCGTCGCCAACCGTAATATTGCAA	ACGAGAGAGATCGCGAATTCATACT	ACGGTTGGCGACGAGCTACATAT

B. Primer sequences for qPCR analysis (5'-3')

Gene name	Forward	Reverse
NvSoxB2a (NvSox1)	CACTGTCCTCCACAAGCTCA	TGAGGCTGCTGATGTCGTAG
NvSoxB2b (NvSox2)	ACGCGTATATGGTATGGTCGA	GGCTTCTTCTACGTAAGGCTGTT
NvSoxB2c (NvSoxB2)	CGATGAATGCGTTCATGGTT	CGCTATAAATGGCTCCTTCTCTT
NvSoxB2d (NvSox3)	AGGACGCAAATCACGTTAAGA	TCGCAGCCCAGAATCTTACTA
NvSoxC	AGTGATGATGATGGTGCTACAGA	TGCTCTCATGCGTGGCTT
NvAshA	ATGGTTCCCACACCTGTTTTAG	TCTCTCTGGCATTTCTTCTCGC
NvAshB	TGTCCAAAGTTGAGACGTTGAGAA	TCTCCGGTAGCTCCATCAATAATG
NvAshC	TCGTAGGCTCCGGCAAACATTAGCA	TGCTAAACGAGTAGTCTTGGAACTCCA
NvAshD	GTAGCGAGGCGTAATGAAAGAGA	ACCGCAACGTCTCCACTTTAGATA
NvArp1	ACTGCAGCGAGAAGTCCTCACTT	TCCTCAAAGGAGAAATTTGGCTCA
NvArp2	AGAGGTCTACCAGACACGGACT	GATGATTCCTCAGGATGAAGTCTC
NvArp3	TGGATCGAAGCCGTCGGCTAA	GTCGACGCAGGGCACCTTCT
NvArp4	ATGGTCTATACACCAAGTAACGA	TGATCTCCGCAGCTTTCTCGA
NvArp5	ACACCACTCCGAGGAGCTTCT	AGACCCTGATGTTCCATCATTGTTAC
NvArp6	GGATGATACTCAAGAGCTAAATTCCC	CTAGGCGGGTCACGTGGTAAGT
NvArp7	ACCACTGTCGCATCAAGCGAG	TCCTTGAATCCCTTGAAGGCGTAT
NvTwist	AGAGGAACTCTGATCAATCCAGT	CGCCGGAGGATCTCTTGT
NvMusashi	ACTACAGCAGAGGGTCTTCGAC	TAGTACGACGTCAACGGCTTT
NvElav1	CCGAGTATGAGCCAAGAGGAT	CCGAGTATGAGCCAAGAGGAT
NvBra	AACCTTTCACCGTCAACGAC	TGACTTGGCTGTCCTCTGTG
NvTcf	CCCAGCTCCTCCTCCTAATC	AATTTGCTGTCTCGCTCCATCAT
NvBmp2/4	ACAGTGACCTCCGCAGAACT	GTCTCCCATTCCAGGCAGTA
NvBmp5-8	GGAGAATACGAGACCTGTTACTG	GACCCATAAGATTCAGTAACTCTTG
NvChordin	AATGCTGTGGTTCCCAGTTC	GCTGAACGCCGATTAGTAGC
NvRfamide	AGTACCATCAGTAGTGATGCTAAAGC	AGCCTTCTTCTGCATCCATGAGAGTT
NvGlwamide	GACGAAGATATGGATGAAACCA	CATAGCCCTATTCCATAAAGATC
NvEf1alpha	GGTTGCCTCTTCGCTTACCACT	CGTTCCTGGCTTTAGGACAC
NvGapdh	GGATGGACCAAGTGCCAAGAAC	GCTTGCCGTTTACCTCAGGAATGA
